# Supplementary material for: Nucleosome positioning shapes cryptic antisense transcription
Source: PLoS Genet. 2026 Mar 13;22(3):e1012078. doi: 10.1371/journal.pgen.1012078 (PMC13075793; doi:10.1371/journal.pgen.1012078)
Supplement: S1 Fig — (DOCX) [file pgen.1012078.s001.docx]

**
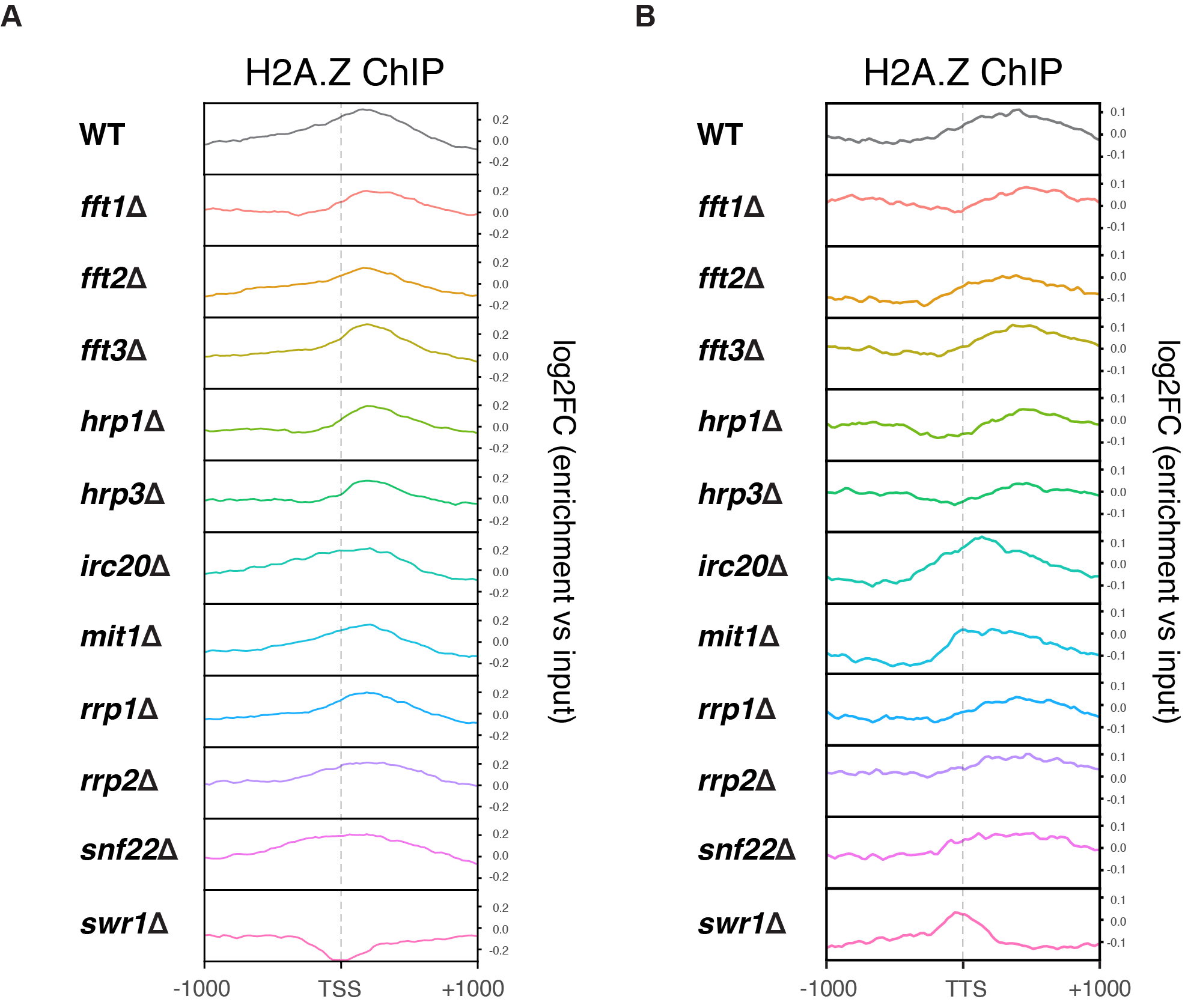
**

**S1 Fig. H2A.Z Enrichment in 11 Nucleosome Remodeler Mutants in Fission Yeast.**

(A) Metagene plots showing ChIP-seq coverage of H2A.Z relative to input across all protein-coding genes in WT, *fft1*Δ, *fft2*Δ, and *fft3*Δ, *hrp1*Δ and *hrp3*Δ, *irc20*Δ, *mit1*Δ, *rrp1*Δ, *rrp2*Δ, *snf22*Δ, and *swr1*Δ mutants. H2A.Z enrichment is plotted relative to the transcription start site (TSS), including 1 kb upstream and downstream.

(B) As in (A), but H2A.Z enrichment is plotted relative to the transcription termination site (TTS) instead.
