# Supplementary material for: Nucleosome positioning shapes cryptic antisense transcription
Source: PLoS Genet. 2026 Mar 13;22(3):e1012078. doi: 10.1371/journal.pgen.1012078 (PMC13075793; doi:10.1371/journal.pgen.1012078)
Supplement: S2 Fig — (DOCX) [file pgen.1012078.s002.docx]

**
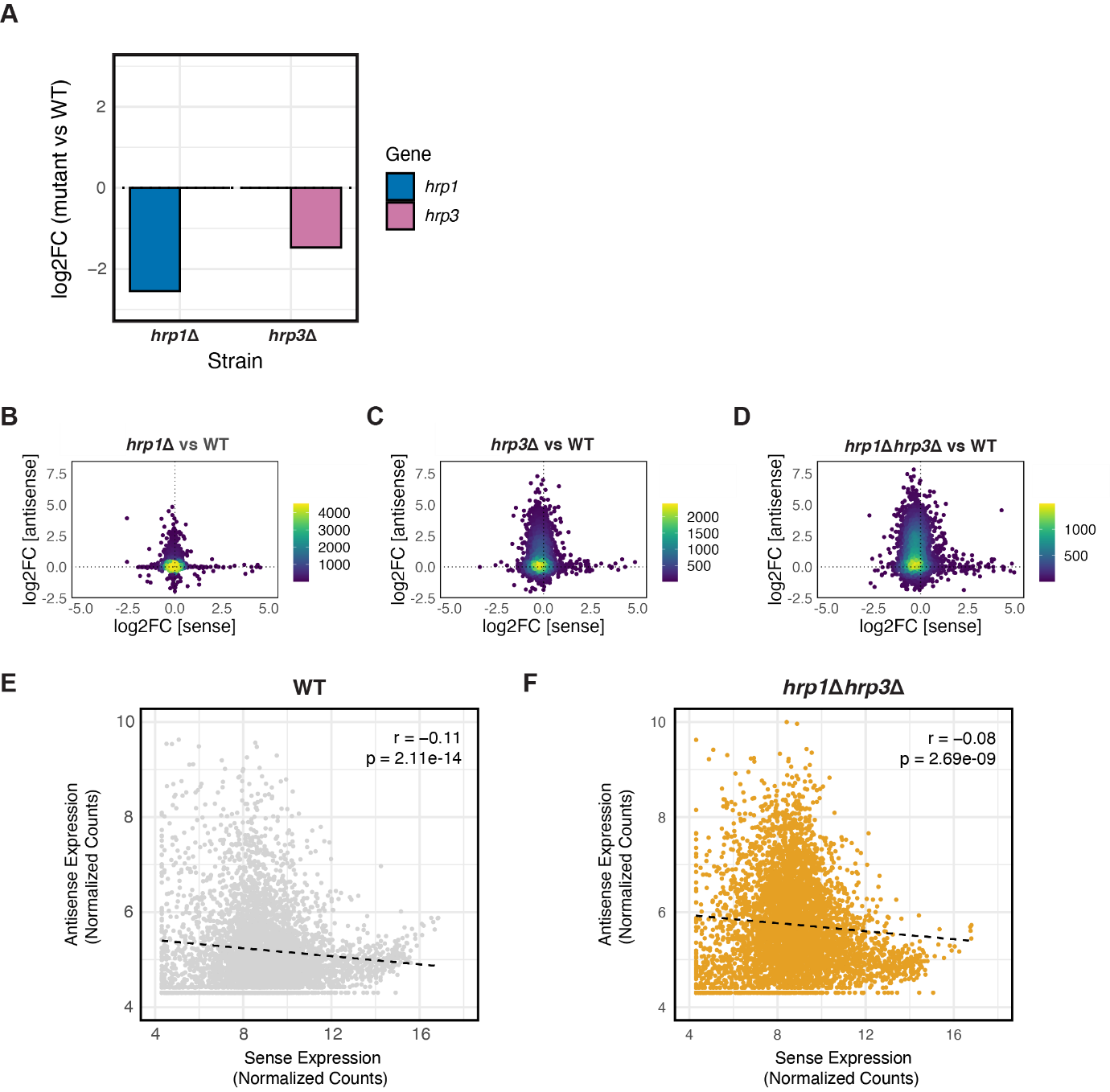
**

**S2 Fig. Analyses of Sense and Antisense Expression in *hrp1*Δ, *hrp3*Δ and *hrp1*Δ*hrp3*Δ.**

(A) Expression levels of *hrp1* and *hrp3* in *hrp3*Δ and *hrp1*Δ mutants, respectively. Expression is shown as log2 fold change (log2FC) relative to WT, based on mRNA-seq data. Values represent the average of three biological replicates.

(B) Density-colored scatterplots of mRNA-seq log2FC values for sense and antisense transcripts in *hrp1*Δ versus WT across all protein-coding genes. Data represents the average of three biological replicates.

(C) As in (B), but for *hrp3*Δ versus WT.

(D) As in (B), but for *hrp1*Δ*hrp3*Δ versus WT.

(E) Scatterplot comparing sense (x-axis) and antisense (y-axis) expression counts across all protein-coding genes in WT. Count data is derived from variance-stabilizing transformation (vst) of raw mRNA-seq counts. Pearson’s correlation and linear regression analysis were performed. Data represents the average of three biological replicates.

(F) As in (E), but for the *hrp1*Δ*hrp3*Δ strain.
