# Supplementary material for: Nucleosome positioning shapes cryptic antisense transcription
Source: PLoS Genet. 2026 Mar 13;22(3):e1012078. doi: 10.1371/journal.pgen.1012078 (PMC13075793; doi:10.1371/journal.pgen.1012078)
Supplement: S3 Fig — (DOCX) [file pgen.1012078.s003.docx]

**
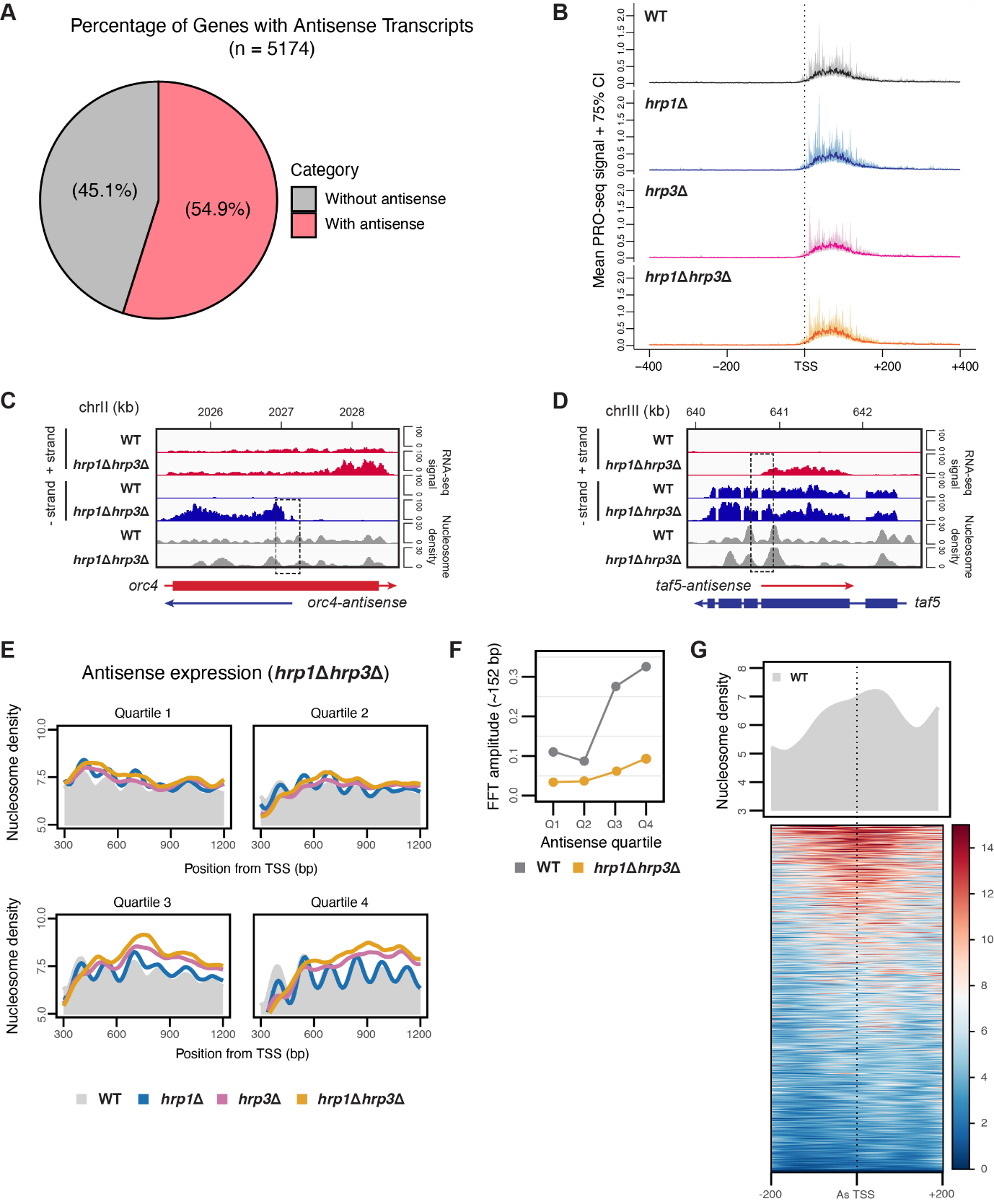
**

**S3 Fig. Additional Analyses of Antisense Transcripts in *hrp1*Δ, *hrp3*Δ and *hrp1*Δ*hrp3*Δ.**

(A) Pie chart showing the proportion of all protein coding genes that have overlapping antisense transcripts (red) and those that do not (grey) in *S. pombe*. The percentages for each category are displayed on the chart.

(B) Metagene representation of mean PRO-seq signals (solid lines) with 75% confidence intervals (shaded regions) for WT, *hrp1Δ*, *hrp3Δ*, and *hrp1Δhrp3Δ* mutants centered on the TSS of the all protein-coding genes in fission yeast. Profiles include 400 bp upstream and downstream of the TSS. Data represent the mean signal from two biological replicates.

(C) Genome browser track of RNA-seq and MNase-seq data for *orc4*, representative of genes with increased antisense transcription in *hrp1Δhrp3Δ* mutants. PRO-seq signals for the + strand (red) and – strand (blue) are displayed. In the gene schematic, solid boxes represent exons, whereas solid lines represent the entire transcript. The dotted box highlights the region where antisense transcription initiates in the *hrp1Δhrp3Δ* mutant, coinciding with disrupted nucleosome positioning.

(D) As in (C), but for the *taf5* gene.

(E) Metaplots of normalized nucleosome density from MNase-seq within the gene body (300 bp to 1200 bp from the TSS) of all protein-coding genes for WT, *hrp1Δ*, *hrp3Δ*, and *hrp1Δhrp3Δ*. Data are stratified into four quartiles based on antisense expression levels (lowest to highest) in the *hrp1Δhrp3Δ* mutant. The WT profile is shown in solid grey, and the plotted data represent the average signal from two biological replicates.

(F) Fourier amplitude at the nucleosomal periodicity (~152 bp) for genes binned into quartiles by antisense transcription in *hrp1*Δ*hrp3*Δ (Q1 = lowest, Q4 = highest), comparing WT and *hrp1*Δ*hrp3*Δ. Points show the mean (fast Fourier transform) FFT amplitude per bin; lines connect quartiles for visualization.

(G) Heatmap of nucleosome density centered on antisense TSSs (vertical dashed line) in WT, shown from −200 to +200 bp. Rows represent antisense loci (ordered by local signal), with warmer colors indicating higher nucleosome density.
