# Supplementary material for: Nucleosome positioning shapes cryptic antisense transcription
Source: PLoS Genet. 2026 Mar 13;22(3):e1012078. doi: 10.1371/journal.pgen.1012078 (PMC13075793; doi:10.1371/journal.pgen.1012078)
Supplement: S4 Fig — (DOCX) [file pgen.1012078.s004.docx]

**
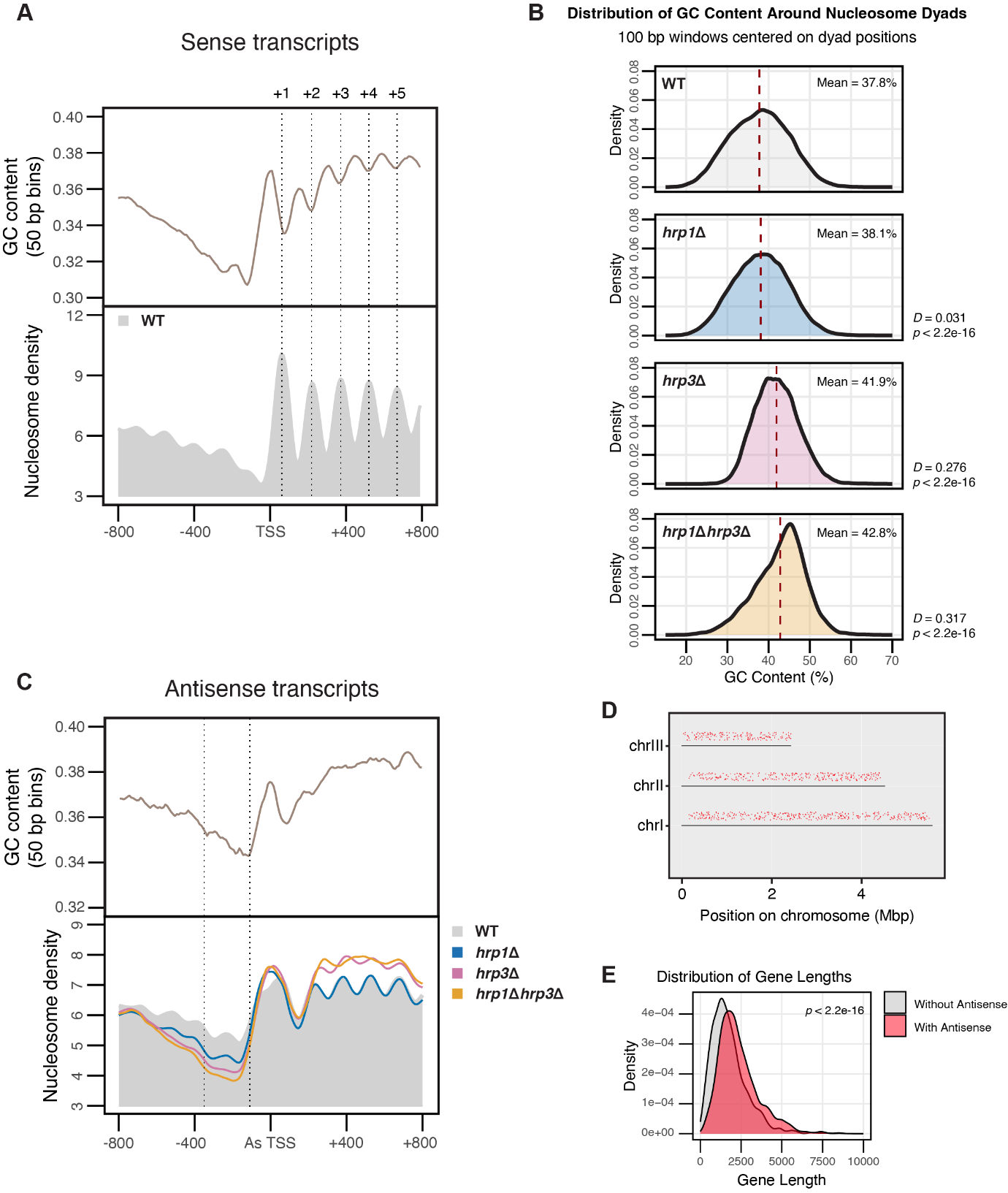
**

**S4 Fig. Loss of *hrp3* is Associated with Depletion of Nucleosome Occupancy over AT-rich Regions.**

(A) (Top) Metaplot of genomic GC content (50 bp bins) across the TSS of all protein coding genes (Bottom) Metaplot of normalized nucleosome density from MNase-seq across the same region. Dotted lines indicate positions of nucleosomes downstream of the TSS. Profiles include 800 bp upstream and downstream of the TSS.

(B) Density histograms of GC content within 100 bp windows centered on all nucleosome dyads in WT, *hrp1Δ, hrp3Δ* and *hrp1Δhrp3Δ*. The dotted line indicates the mean GC content. The Kolmogorov-Smirnov test was used to compare the distributions for statistical significance between WT and each mutant, reported are the *D*-statistic and *p*-value.

(C) (Top) Metaplot of genomic GC content (50 bp bins) across the antisense (As) TSS of 4,678 antisense transcripts. (Bottom) Metaplot of normalized nucleosome density from MNase-seq across the same region (as shown in Fig. 3D). The black dotted line indicates the region directly upstream of the As TSS where nucleosome density is decreased in *hrp1Δ, hrp3Δ* and *hrp1Δhrp3Δ*. Profiles include 800 bp upstream and downstream of the As TSS.

(D) Distribution of antisense expression across chromosomes I, II, and III in the *hrp1Δhrp3Δ* mutant. Each red dot represents a gene with an antisense transcript.

(E) The density plot shows the distribution of gene lengths for two groups: genes with antisense transcripts (red) and genes without antisense transcripts (grey). The Wilcoxon rank sum test was used to compare the two distributions for statistical significance.
