# Supplementary material for: Nucleosome positioning shapes cryptic antisense transcription
Source: PLoS Genet. 2026 Mar 13;22(3):e1012078. doi: 10.1371/journal.pgen.1012078 (PMC13075793; doi:10.1371/journal.pgen.1012078)
Supplement: S5 Fig — (DOCX) [file pgen.1012078.s005.docx]

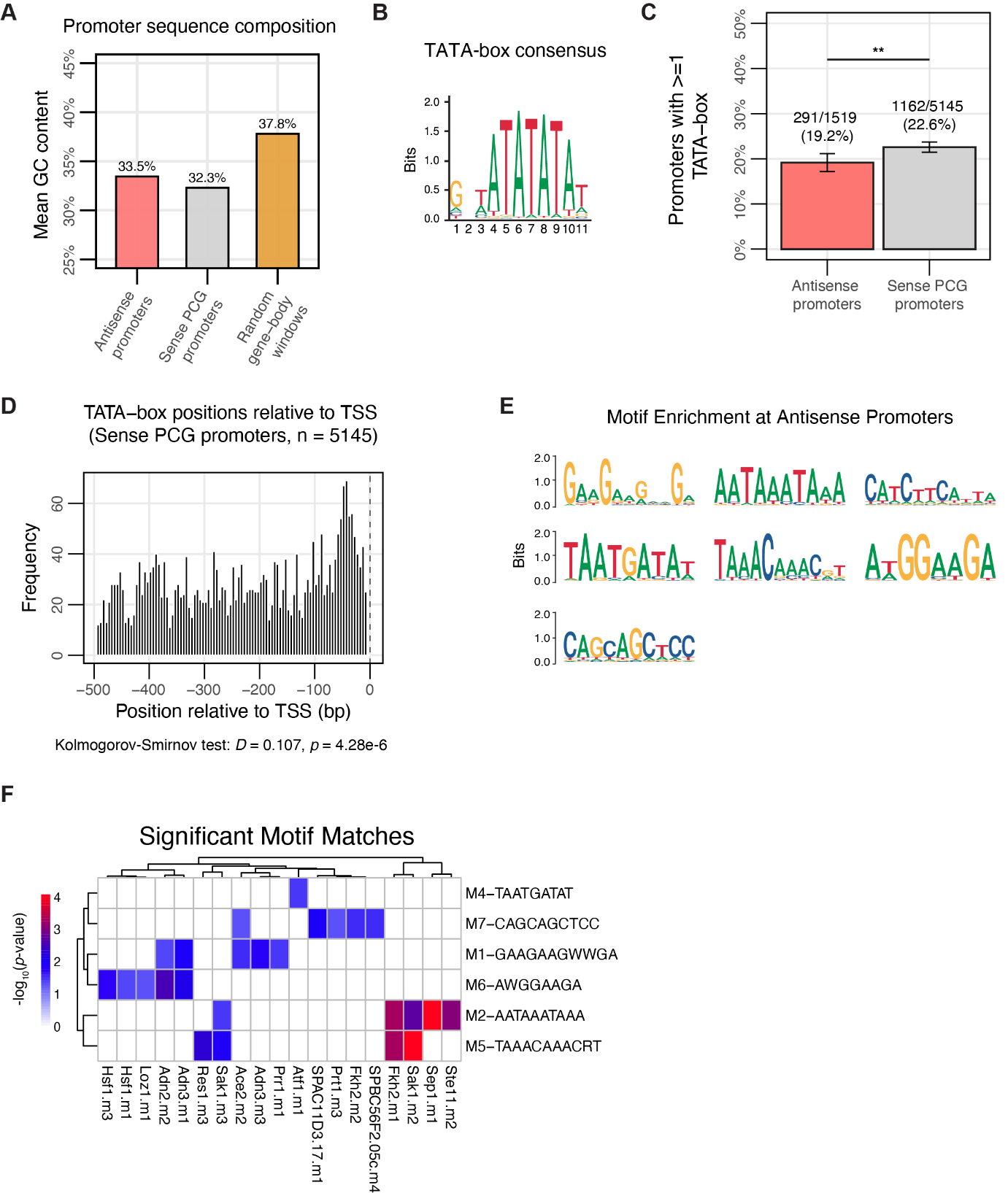


**S5 Fig. Characteristics of Cryptic Antisense Promoters Identified in** ***hrp3*Δ and *hrp1*Δ*hrp3*Δ.**

(A) GC content comparison between antisense promoters, promoters of sense protein-coding genes (PCGs), and random gene-body windows. Bars show the mean GC fraction per 500 bp window (promoter windows are upstream of the TSS; gene-body windows are randomly sampled). GC is computed per sequence from A/C/G/T counts and plotted as percentages.

(B) Consensus TATA-box motif in yeast (TBP-binding), retrieved from the JASPAR CORE Fungi (redundant) database, entry MA0386.2. Shown as a sequence logo generated from the position weight matrix (PWM), corresponding to the TATAWAWR consensus.

(C) Fraction of promoters classified as TATA-box–positive. Bars show the proportion of promoters with at least one TATA-box hit (FIMO, PWM as in (B), *p* ≤ 1e-4) for antisense promoters versus all sense PCG promoters. Error bars indicate 95% binomial confidence intervals. Statistical analysis was performed using Fisher’s exact test. Asterisks indicate statistical significance: p < 0.05 (*), p < 0.01 (**), p < 0.001 (***).

(D) Genomic positions of TATA-box hits relative to the antisense TSS for antisense promoters. Histogram shows the distribution of TATA PWM match centers (5 bp bins) within −500 to −1 bp upstream of the TSS (FIMO, *p* ≤ 1e-4). The dashed vertical line marks the TSS (0 bp). Positional distributions were compared with (Fig. 3G) using a two-sample Kolmogorov–Smirnov (KS) test; reported p-values are from the KS test.

(E) De novo motif discovery at 500 bp regions upstream of the As TSS. Motif discovery was performed using STREME from the MEME toolbox. Motifs shown here have passed the significance threshold of *p* < 0.05.

(F) Matching and comparison of the seven motifs identified in (E) against the *S. pombe* transcription factor (TF) atlas from Skribbe et al. Analysis was performed using TOMTOM from the MEME toolbox. The color on the heatmap represents significance, and all colored comparisons have passed a significance threshold of *p* < 0.05. TFs with more than one reported motif are denoted with a numbered suffix (e.g. Fkh2.m1 and Fkh2.m2).
