# Supplementary material for: Nucleosome positioning shapes cryptic antisense transcription
Source: PLoS Genet. 2026 Mar 13;22(3):e1012078. doi: 10.1371/journal.pgen.1012078 (PMC13075793; doi:10.1371/journal.pgen.1012078)
Supplement: S6 Fig — (DOCX) [file pgen.1012078.s006.docx]

**
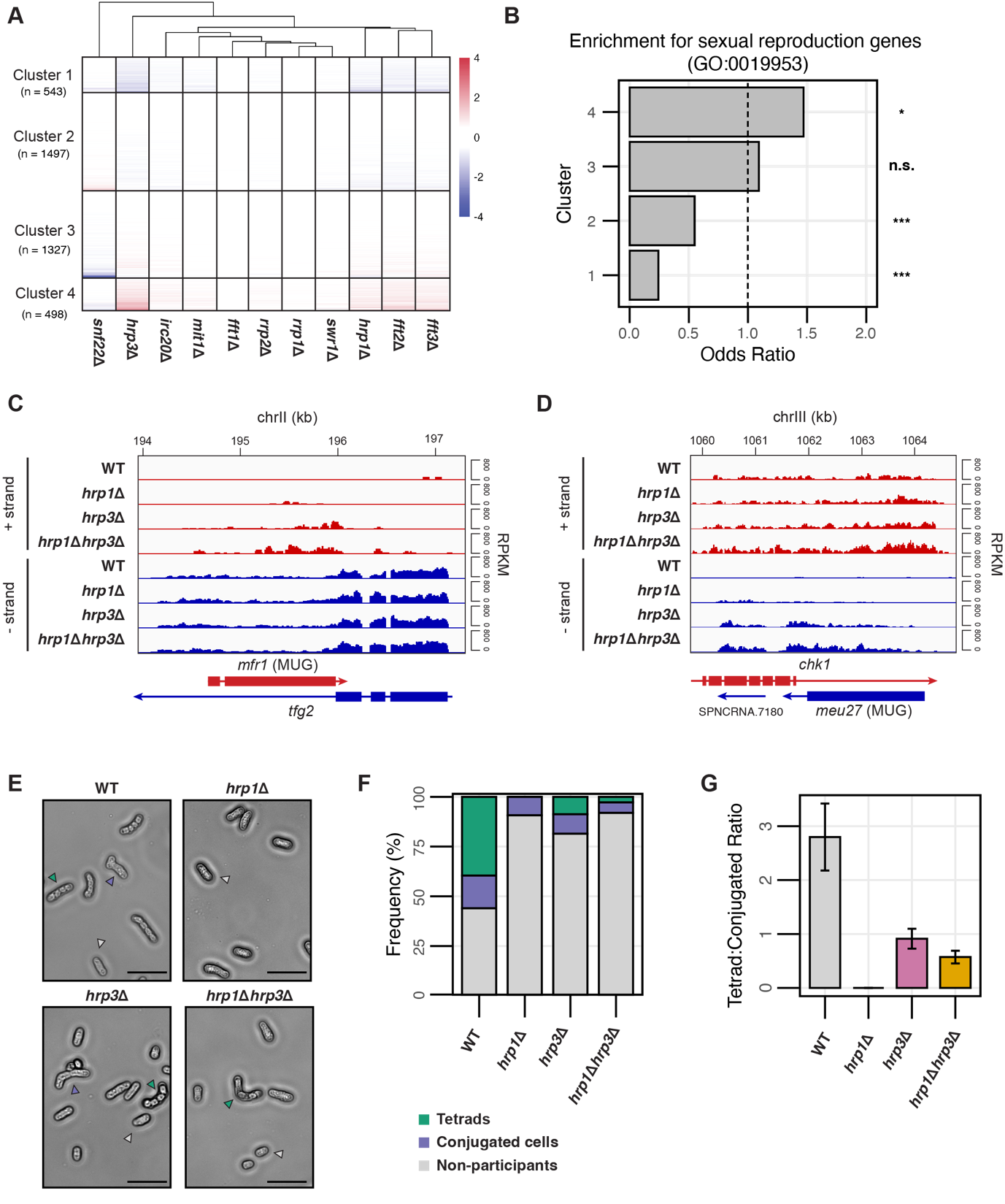
**

**S6 Fig. Further Analyses on MUGs and Convergent Nested Genes in Fission Yeast.**

(A) Heatmap of significantly differentially expressed protein-coding genes (adj p-value < 0.05, DESeq2) in 11 nucleosome remodeler deletion mutants relative to WT, based on mRNA-seq data. The color scale represents log2 fold change (log2FC) in gene expression for each mutant compared to WT. Genes are stratified into four clusters based on their expression patterns.

(B) Enrichment analysis of the differentially expressed genes from (A) across the four clusters for the sexual reproduction GO term (GO:0019953). The dashed line indicates an odds ratio of 1. Statistical analysis was performed using Fisher’s exact test. Asterisks indicate statistical significance: p < 0.05 (*), p < 0.01 (**), p < 0.001 (***).

(C) Genome browser track of mRNA-seq data for the *mfr1*/*tfg2* nested gene pairs in WT, *hrp1*Δ*, hrp3*Δ *and hrp1*Δ*hrp3*Δ. Tracks are separated into the + strand (red) and – strand (blue), with values shown in RPKM. The MUG within the pair is denoted in brackets.

(D) As in (C), but for the *chk1*/ *meu27* nested gene pair.

(E) Representative phase-contrast images 24 h after meiotic induction for WT, *hrp1*Δ*, hrp3*Δ *and hrp1*Δ*hrp3*Δ.. Scale bars represent 20 μm. Arrowheads indicate tetrads (green), conjugated pairs (purple) and non‑participants (gray).

(F) Distribution of cell categories for WT, *hrp1*Δ*, hrp3*Δ *and hrp1*Δ*hrp3*Δ 24 h after meiotic induction. Cell counts represent the mean across 6 microscopic fields for each genotype, with total counts ranging from 39-193 cells for each field.

(G) Bar plot showing the ratio between tetrads and conjugated cells for WT, *hrp1*Δ*, hrp3*Δ *and hrp1*Δ*hrp3*Δ. Error bars represent the mean ± SEM.
