# Supplementary material for: Nucleosome positioning shapes cryptic antisense transcription
Source: PLoS Genet. 2026 Mar 13;22(3):e1012078. doi: 10.1371/journal.pgen.1012078 (PMC13075793; doi:10.1371/journal.pgen.1012078)
Supplement: S7 Fig — (DOCX) [file pgen.1012078.s007.docx]

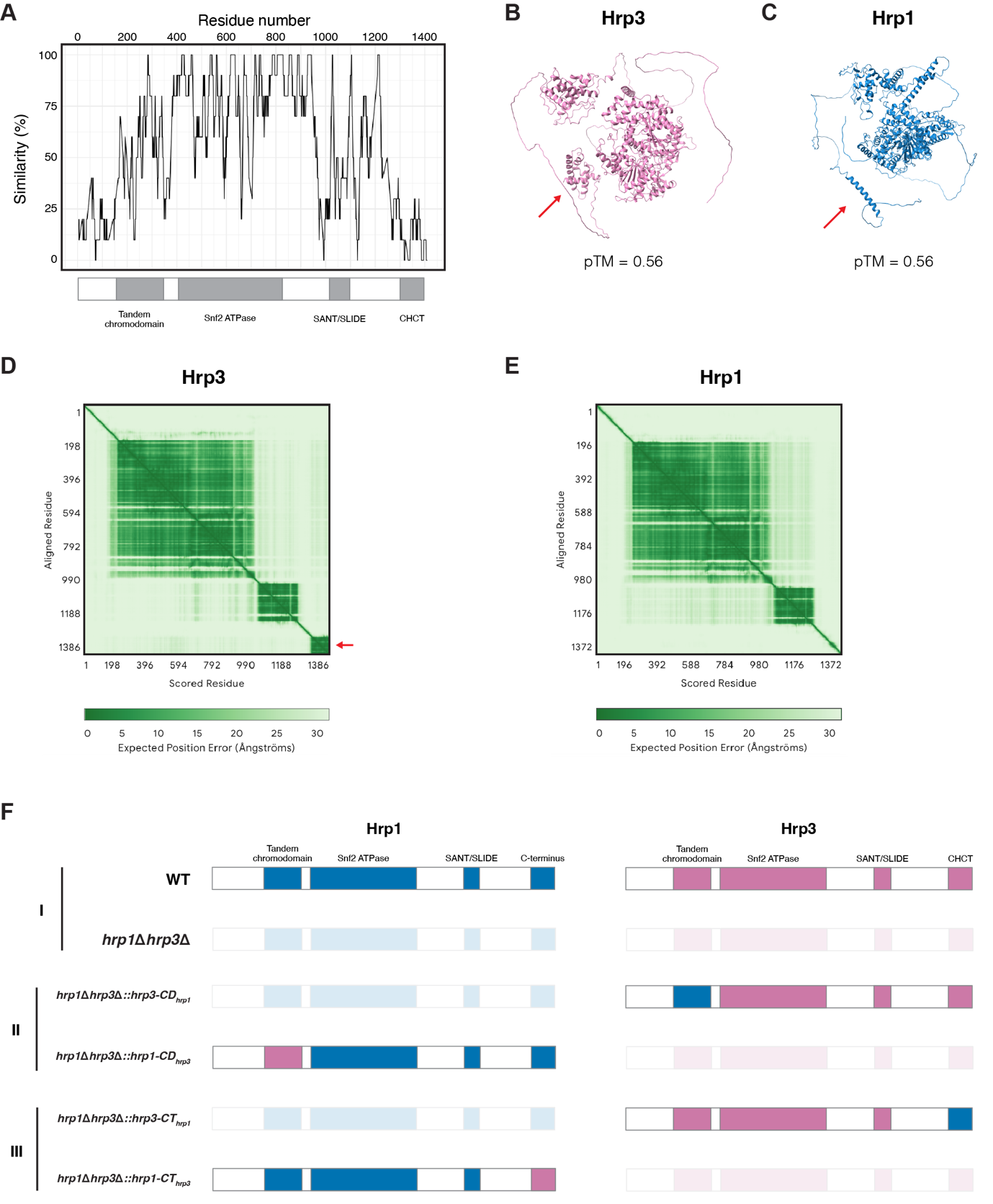


**S7 Fig. Comparative Domain Analysis of Hrp1 and Hrp3.**

(A) Sliding window analysis of protein sequence similarity between Hrp1 and Hrp3. Sequence similarity was calculated using a sliding window of 25 residues after multiple sequence alignment (MSA) with the msa R package (default settings). Each point represents the similarity score for a specific window, plotted against the central residue position. Gaps in the alignment were excluded to ensure accurate similarity calculations. Protein domains are annotated below the plot.

(B) AlphaFold3-predicted structure of Hrp3. The red arrow marks the C-terminus. The pTM value represents the Predicted Template Modelling (pTM) score.

(C) AlphaFold3-predicted structure of Hrp1. The red arrow indicates the CHCT domain. The pTM value represents the Predicted Template Modelling (pTM) score.

(D) Predicted Aligned Error (PAE) plot from AlphaFold3 for Hrp3, showing the expected position error for each residue.

(E) PAE plot for Hrp1, as in (D). The red arrow highlights the expected positional error for the CHCT domain.

(F) Schematic representation of protein domain configurations in Hrp1 and Hrp3 for wild-type (WT) and mutant strains. Mutants include *hrp1*Δ*hrp3*Δ, *hrp1*Δ*hrp3*Δ*::hrp3-CD_hrp1_*, *hrp1*Δ*hrp3*Δ*::hrp1-CD_hrp3_*, *hrp1*Δ*hrp3*Δ*::hrp3-CT_hrp1_*, and *hrp1*Δ*hrp3*Δ*::hrp1-CT_hrp3_*. CD stands for chromodomain, while CT stands for C-terminus. Domain swaps are indicated by color changes, and greyed-out proteins represent their absence in the respective mutants.
