# Supplementary material for: Nucleosome positioning shapes cryptic antisense transcription
Source: PLoS Genet. 2026 Mar 13;22(3):e1012078. doi: 10.1371/journal.pgen.1012078 (PMC13075793; doi:10.1371/journal.pgen.1012078)
Supplement: S8 Fig — (DOCX) [file pgen.1012078.s008.docx]

**
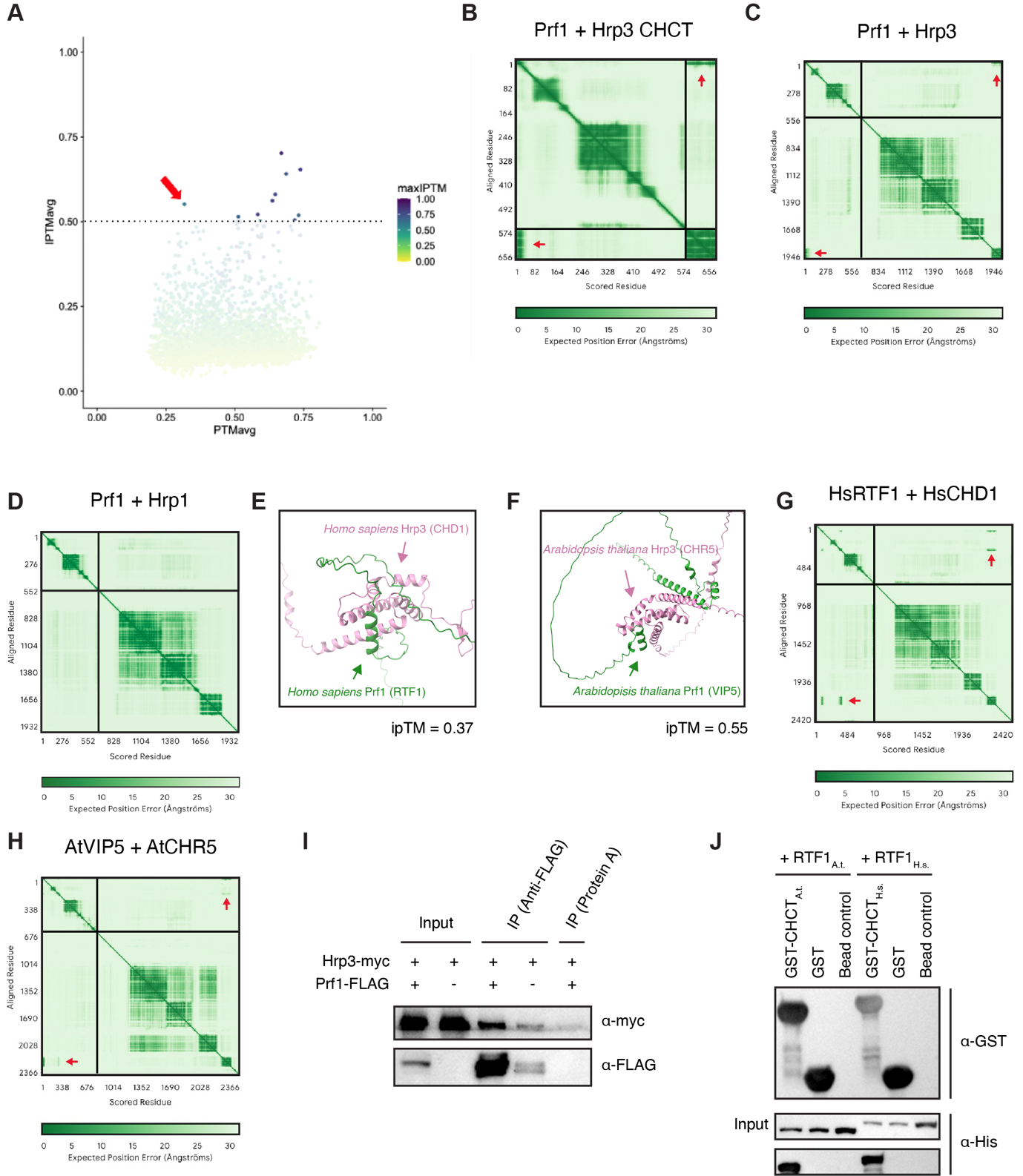
**

**S8 Fig. AlphaFold-predicted Interactions Between Prf1 and the Hrp3 CHCT Domain Across Species.**

(A) Scatterplot of the average Interface Predicted Template Modelling (ipTM) score versus the average Predicted Template Modelling (pTM) score from an *in-silico* screen using AlphaFold2 Multimer. The screen was performed on the Hrp3 CHCT domain with a set of 2,692 genes known to localize to the *S. pombe* nucleus. The dotted line indicates an average ipTM score cutoff of 0.5. The red arrow highlights Prf1.

(B) Predicted Aligned Error (PAE) plot from AlphaFold3 showing the predicted binding between Prf1 and the CHCT domain of Hrp3. Red arrows indicate the predicted interface with low expected positional error between the N-terminus of Prf1 and the CHCT domain.

(C) As in (B), but showing the predicted binding between Prf1 and full-length Hrp3. Red arrows indicate the predicted interface with low expected positional error between the N-terminus of Prf1 and the C-terminus of Hrp3.

(D) As in (B), but showing the predicted binding between Prf1 and full-length Hrp1.

(E) AlphaFold3-predicted structure of the interaction between the N-terminus of *Homo sapiens* Prf1 (RTF1) and the CHCT domain of *Homo sapiens* Hrp3 (CHD1).

(F) As in (E), but showing the interaction between the N-terminus of *Arabidopsis thaliana* Prf1 (VIP5) and the CHCT domain of *Arabidopsis thaliana* Hrp3 (CHR5).

(G) Predicted Aligned Error (PAE) plot from AlphaFold3 showing the predicted binding between *Homo sapiens* RTF1 and CHD1. Red arrows indicate the predicted interface with low expected positional error between the N-terminus of RTF1 and the C-terminus of CHD1.

(H) As in (F), but showing the predicted binding between *Arabidopsis thaliana* VIP5 and CHR5. Red arrows indicate the predicted interface with low expected positional error between the N-terminus of VIP5 and the C-terminus of CHR5.

(I) Endogenous co‑immunoprecipitation of Hrp3 with Prf1 from *S. pombe*. Whole‑cell extracts (Input) were prepared from strains expressing Prf1‑FLAG and Hrp3‑Myc from their native loci, alongside an otherwise isogenic no-FLAG control strain. Anti-FLAG immunoprecipitations (IP) were analyzed by immunoblotting with anti-Myc to detect co-precipitating Hrp3-Myc (top) and anti-FLAG to confirm specific recovery of Prf1-FLAG (bottom). Protein A–agarose beads were included as a matrix control.

(J) Western blot showing anti-GST and anti-His signals for GST-pulldowns of GST-CHCT and wild-type His-Prf1/RTF1 from either *Arabidopsis thaliana* (A.t.) or *Homo sapiens* (H.s.).
