# Supplementary material for: Nucleosome positioning shapes cryptic antisense transcription
Source: PLoS Genet. 2026 Mar 13;22(3):e1012078. doi: 10.1371/journal.pgen.1012078 (PMC13075793; doi:10.1371/journal.pgen.1012078)
Supplement: S9 Fig — (DOCX) [file pgen.1012078.s009.docx]

**
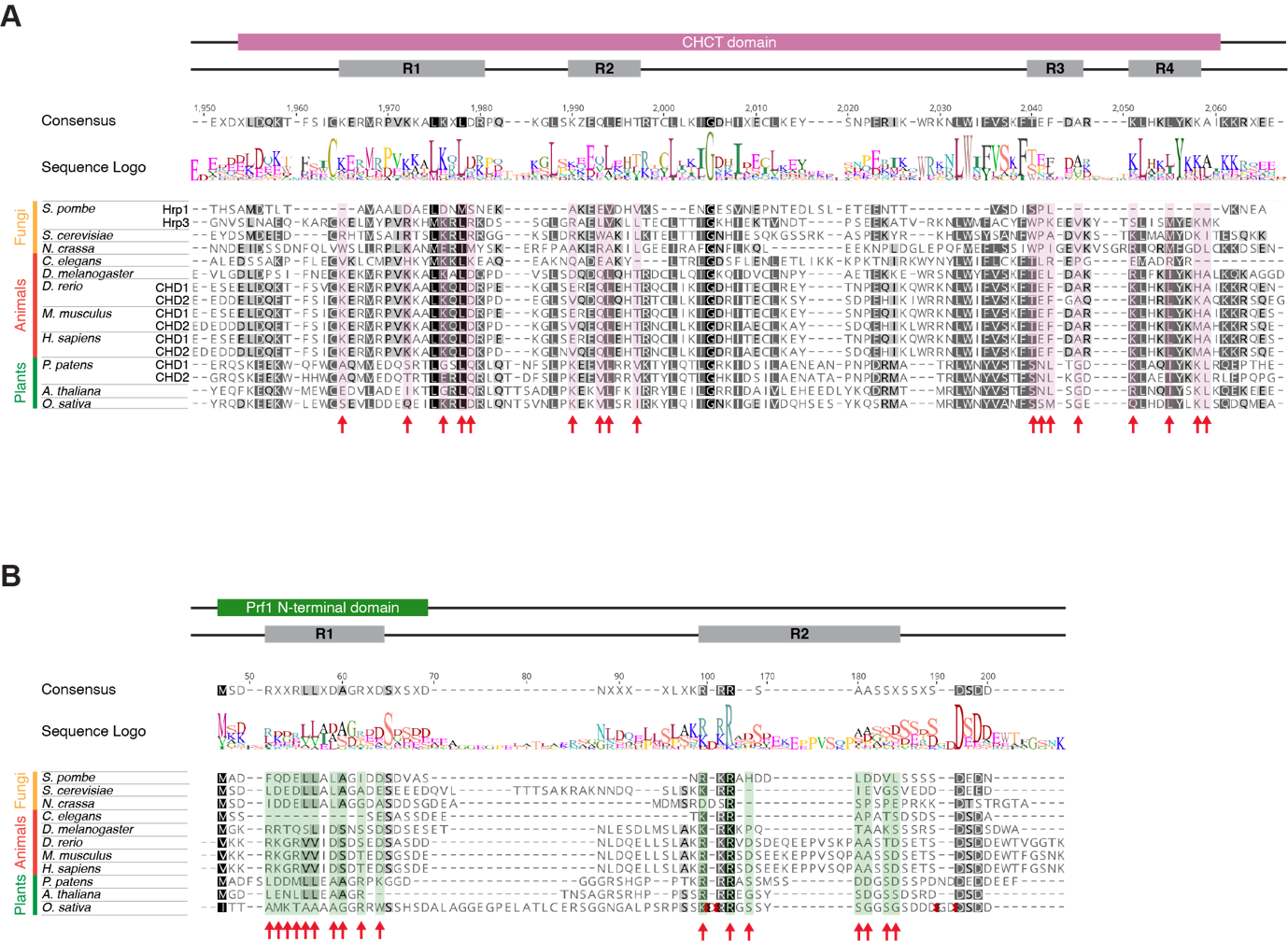
**

**S9 Fig. Interacting Residues Between Hrp3 and Prf1 are Conserved Across Eukaryotes.**

(A) Multiple sequence alignment (MSA) of the CHCT domain of Hrp3 across 11 eukaryotic species, performed using Multiple Alignment using Fast Fourier Transform (MAFFT). For species with two paralogs, both copies are included. Residues are shaded in increasing intensities of black based on their degree of conservation across species. Residues predicted by AlphaFold3 to interact with Prf1 are annotated with red arrows and highlighted in pink, corresponding to four regions (R1, R2, R3, and R4). Hrp1 is included as a comparison, despite lacking the CHCT domain. Species used in the analysis were *Schizosaccharomyces pombe, Saccharomyces cerevisiae, Neurospora crassa, Caenorhabditis elegans, Drosophila melanogaster, Danio rerio, Mus musculus, Homo sapiens, Physcomitrella patens, Arabidopsis thaliana,* and *Oryza sativa*.

(B) As in (A), but showing the MSA results for the N-terminal region of Prf1. Residues predicted by AlphaFold3 to interact with Hrp3 are annotated with red arrows and highlighted in green, corresponding to two regions (R1 and R2).
