# Supplementary material for: Nucleosome positioning shapes cryptic antisense transcription
Source: PLoS Genet. 2026 Mar 13;22(3):e1012078. doi: 10.1371/journal.pgen.1012078 (PMC13075793; doi:10.1371/journal.pgen.1012078)
Supplement: S10 Fig — (DOCX) [file pgen.1012078.s010.docx]

**
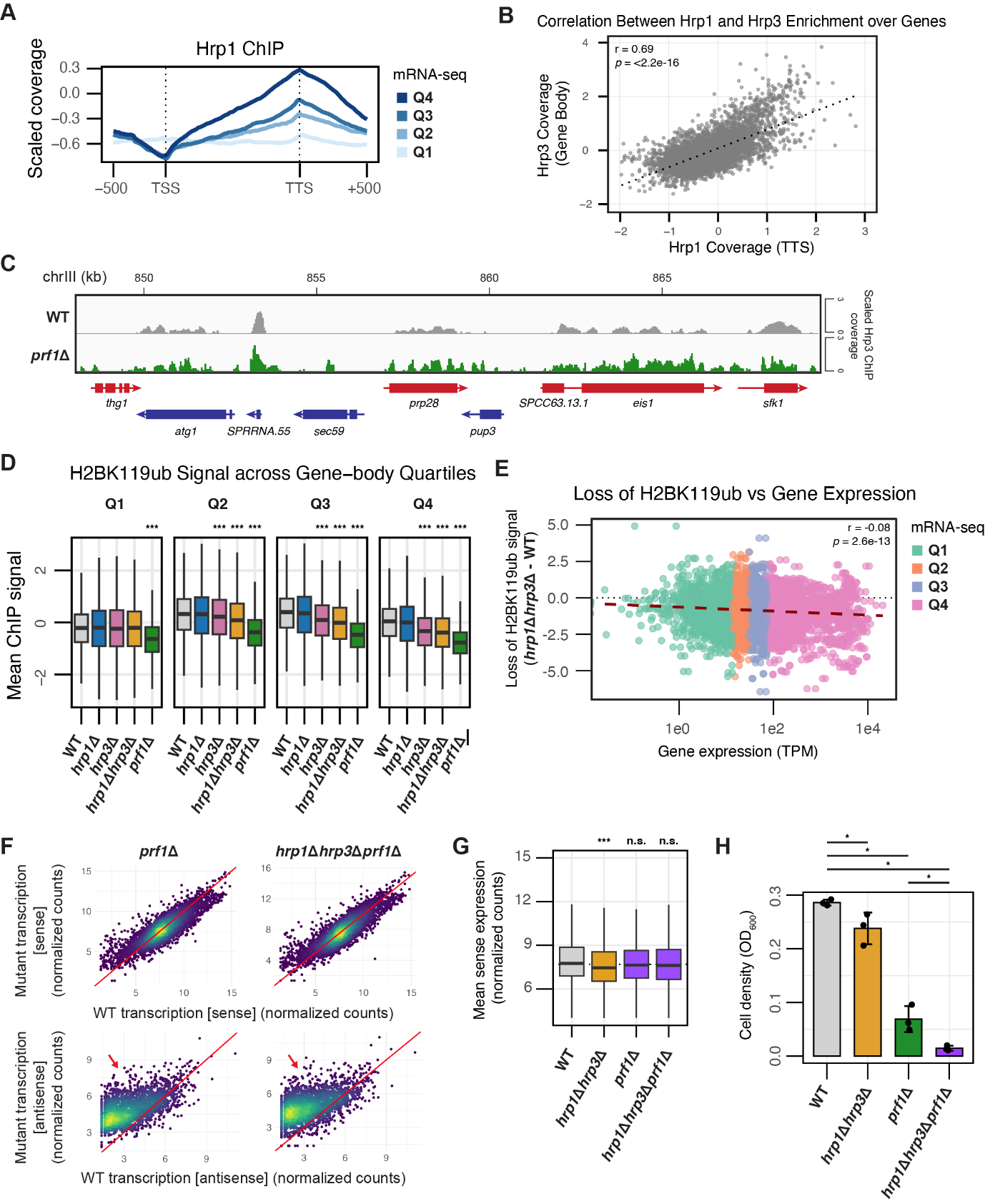
**

**S10 Fig. Additional Analyses of *prf1* Mutants.**

(A) Metagene plot of the ChIP-seq coverage relative to input for Hrp1-myc in WT across all protein coding genes. Genes were divided into four quartiles based on expression in WT. Quartiles are numbered by expression level, with Q1 being the lowest and Q4 being the highest.

(B) Scatterplot showing the correlation between the ChIP-seq coverage relative to input for Hrp3 in the gene body versus the ChIP-seq coverage relative to input for Hrp1 at the TTS for all protein coding genes. Each point represents a single gene, with the x-axis corresponding to the mean Hrp1 signal in a 500 bp region centered on the TTS and the y-axis corresponding to the mean Hrp3 signal across the gene body (from TSS to TTS). The black dotted line represents the linear regression fit. The Pearson correlation coefficient (r) and p-value (p) are displayed on the plot.

(C) Genome browser view of Hrp3 ChIP–seq coverage (RPGC‑normalized) across a representative region on chromosome II. Tracks show WT (gray) and *prf1*Δ (green) Hrp3 profiles aligned to gene annotations (red, forward strand; blue, reverse strand).

(D) Boxplots of mean H2BK119ub ChIP signal within gene bodies in WT, *hrp1*Δ, *hrp3*Δ, *hrp1*Δ *hrp3*Δ, and *prf1*Δ, stratified by gene‑body quartiles (Q1–Q4, lowest to highest signal in WT). Statistical analysis was performed using Welch’s two-sample t-test versus WT. Asterisks indicate statistical significance: p < 0.05 (*), p < 0.01 (**), p < 0.001 (***).

(E) Scatterplot showing the correlation between the loss of H2BK119ub in *hrp1*Δ*hrp3*Δ*prf1*Δ versus WT and gene expression in WT across all protein-coding genes. The dashed line represents the linear regression. Points are color-coded by quartiles of gene expression. The dotted line at y = 0 indicates no loss of H2Bub signal. The Spearman correlation coefficient and p-value are displayed on the plot.

(F) Density-colored scatterplots comparing transcription in mutants (*prf1*Δ and *hrp1*Δ*hrp3*Δ*prf1*Δ) to WT for sense transcripts (top row) and antisense transcripts (bottom row) across all protein-coding genes. Count data are derived from vst-transformed raw mRNA-seq counts. Red arrows highlight elevated antisense transcription in mutants relative to WT.

(G) Boxplot of variance-stabilizing transformation (vst)-normalized sense expression counts for WT, *hrp1*Δ*hrp3*Δ, *prf1*Δ, and *hrp1*Δ*hrp3*Δ*prf1*Δ. The dotted line represents the median sense expression in WT. Statistical analysis was performed using ANOVA with Tukey’s Honestly Significant Difference (HSD) test. Asterisks indicate statistical significance: p < 0.05 (*), p < 0.01 (**), p < 0.001 (***).

(H) Bar plot of cell density (OD600; mean ± s.e.m., n = 3 biological replicates) for WT, *hrp1*Δ*hrp3*Δ, *prf1*Δ, and *hrp1*Δ*hrp3*Δ*prf1*Δ grown in YES at 32 C for 15 h. Black dots show individual replicates. Statistical analysis was performed using pairwise comparisons versus WT with two‑sided t‑tests. Asterisks indicate statistical significance: p < 0.05 (*), p < 0.01 (**), p < 0.001 (***).
