# Supplementary material for: Nucleosome positioning shapes cryptic antisense transcription
Source: PLoS Genet. 2026 Mar 13;22(3):e1012078. doi: 10.1371/journal.pgen.1012078 (PMC13075793; doi:10.1371/journal.pgen.1012078)
Supplement: S11 Fig — (DOCX) [file pgen.1012078.s011.docx]

**
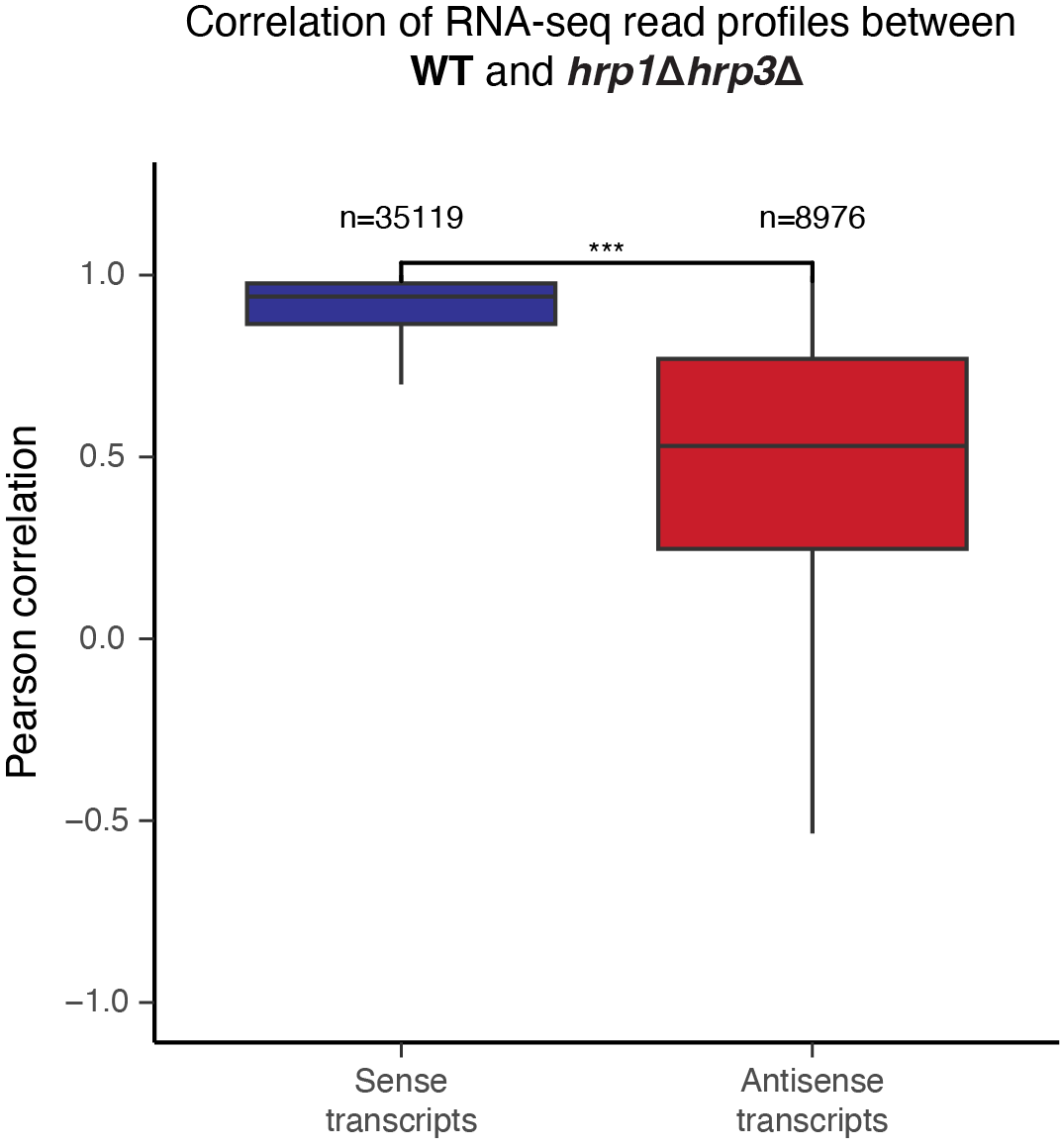
**

**S11 Fig. Analysis of cryptic sense transcription in *hrp1*Δ*hrp3*Δ.**

Correlation of RNA‑seq read profiles between WT and *hrp1*Δ*hrp3*Δ. For each annotated transcript, strand‑specific RNA‑seq coverage was computed across the gene body and Pearson correlation coefficients were calculated between WT and the double mutant. Boxplots show the distribution of per‑gene correlations for sense (blue; n = 5,109) and antisense (red; n = 8,976) transcripts. Statistical analysis was performed using a two‑sided Wilcoxon rank‑sum test. Asterisks indicate statistical significance: p < 0.05 (*), p < 0.01 (**), p < 0.001 (***).
