# Supplementary material for: Nucleosome positioning shapes cryptic antisense transcription
Source: PLoS Genet. 2026 Mar 13;22(3):e1012078. doi: 10.1371/journal.pgen.1012078 (PMC13075793; doi:10.1371/journal.pgen.1012078)
Supplement: S1 Note — (DOCX) [file pgen.1012078.s012.docx]

**Supplementary Notes**

**S1 Note. Transcription of cryptic sense isoforms in *hrp1*Δ*hrp3*Δ**

To assess whether *hrp1*Δ*hrp3*Δ shows increased transcription of cryptic sense isoforms, we analyzed orthogonal datasets from long-read transcriptomics and mRNA-seq to assess intragenic sense initiation. We operated according to the rationale that if substantial sense cryptic initiation were occurring within gene bodies in the *hrp1*Δ*hrp3*Δ, it should alter the intra‑gene distribution of reads (i.e., the profile shape) across affected loci compared to WT, even when total gene‑level counts remain similar. Such changes would appear as detectable shifts or distortions in binned coverage profiles.

We generated a custom GTF of sense isoforms from Iso‑Seq long reads (retaining transcripts overlapping curated PomBase genes) and, for WT and *hrp1*Δ*hrp3*Δ, merged strand‑specific mRNA‑seq replicates, computed sense‑strand coverage along each Iso‑Seq transcript (TSS→TES) in 50‑bp bins, and correlated per‑transcript binned profiles between genotypes; the same analysis was performed for antisense coverage and distributions were compared by Wilcoxon tests (**Fig. S11**). Sense coverage shapes were highly concordant between WT and *hrp1*Δ*hrp3*Δ (median Pearson r ≈ 0.95), whereas antisense profiles showed strong redistribution (median r ≈ 0.53; Wilcoxon P < 1×10^−16; rank‑biserial effect size = 0.663). These analyses argue against widespread, high‑level cryptic sense initiation that would measurably distort sense gene‑body profiles, in contrast to the pronounced antisense activation reported here; accordingly, we do not claim genome‑wide cryptic sense initiation in this study.
