# Supplementary material for: Nucleosome positioning shapes cryptic antisense transcription
Source: PLoS Genet. 2026 Mar 13;22(3):e1012078. doi: 10.1371/journal.pgen.1012078 (PMC13075793; doi:10.1371/journal.pgen.1012078)
Supplement: S1 Table — (DOCX) [file pgen.1012078.s013.docx]

**S1 Table**. *De novo* motif discovery at cryptic antisense promoters. Motif discovery was performed using STREME, showing significant (*p*-value < 0.05) results for the 500 bp regions upstream of the As TSS (n = 1519) against the *S. pombe* transcription factor atlas from Skribbe et al.

| Motif ID | Motif sequence | *p*-value | No. of sites |
| --- | --- | --- | --- |
| M1 | GAAGAAGWWGA | 5.74e-04 | 758 |
| M2 | AATAAATAAA | 7.3e-03 | 651 |
| M3 | CATCTTCATTA | 1.0e-02 | 779 |
| M4 | TAATGATAT | 1.5e-02 | 536 |
| M5 | TAAACAAACRT | 1.8e-02 | 261 |
| M6 | AWGGAAGA | 3.4e-02 | 178 |
| M7 | CAGCAGCTCC | 4.0e-02 | 138 |
