# Supplementary material for: Nucleosome positioning shapes cryptic antisense transcription
Source: PLoS Genet. 2026 Mar 13;22(3):e1012078. doi: 10.1371/journal.pgen.1012078 (PMC13075793; doi:10.1371/journal.pgen.1012078)
Supplement: S2 Table — (DOCX) [file pgen.1012078.s014.docx]

**S2 Table**. Early, middle and late MUGs upregulated in *hrp3*Δ*.*

| Gene ID | Name | Description |
| --- | --- | --- |
| **Early MUGs** | | |
| SPAC17A5.18c | rec25 | meiotic recombination protein Rec25 |
| SPAC25G10.04c | rec10 | meiotic recombination protein Rec10 |
| SPAC6C3.05 | meu43 | Schizosaccharomyces specific protein Meu43 |
| SPAC8E11.03c | dmc1 | RecA family ATPase Dmc1 |
| SPBC21B10.12 | rec6 | meiotic recombination protein Rec6 |
| SPBC31F10.05 | mug37 | conserved fungal protein, OB fold, predicted role in DNA metabolism |
| SPCC1620.04c | fzr3 | meiotic fizzy-related APC coactivator Fzr3 |
| **Middle MUGs** | | |
| SPAC1250.02 | mug95 | Schizosaccharomyces specific protein Mug95 |
| SPAC1610.03c | crp79 | poly(A) binding protein Crp79 |
| SPAC16A10.08c | mug74 | Schizosaccharomyces specific protein Mug74 |
| SPAC16E8.05c |  | Schizosaccharomyces specific protein Mde1 |
| SPAC1A6.06c | meu31 | Schizosaccharomyces specific protein Meu31 |
| SPAC212.02 |  | Schizosaccharomyces pombe specific protein |
| SPAC22F3.02 | atf31 | DNA-binding transcription factor Atf31 |
| SPAC22F3.04 | mug62 | implicated in acetyl-CoA biosynthesis and diacylglycerol metabolism |
| SPAC24C9.15c | spn5 | meiotic septin Spn5 |
| SPAC25H1.09 | mde5 | alpha-amylase homolog Mde5 |
| SPAC343.07 | mug28 | RNA-binding protein Mug28, implicated in mRNA processing |
| SPAC4G9.05 | mpf1 | cytoplasmic meiotic pumilio family RNA-binding protein Mpf1 |
| SPAC6B12.06c | rrg9 | mitochondrial ribosome assembly protein Rrg9 |
| SPAC6C3.07 | mug68 | Schizosaccharomyces specific protein Mug68 |
| SPAC6G10.06 | tda3 | FAD-dependent amino acid oxidase involved in late endosome to Golgi transport Tda3 |
| SPAC8F11.05c | mug130 | Schizosaccharomyces specific protein Mug130 |
| SPAC977.06 |  | S. pombe specific DUF999 family protein 3 |
| SPAPB17E12.09 |  | conserved protein, expressed during meiotic cell cycle, possibly related to metazoan RAB11FIP4 family |
| SPBC1198.12 | mfr1 | meiotic APC activator Mfr1 |
| SPBC146.11c | mug97 | meiotically upregulated gene Mug97 |
| SPBC1685.06 | cid11 | poly(A) polymerase Cid11, terminal uridylyl transferase |
| SPBC16A3.13 | meu7 | alpha-amylase homolog Aah4 |
| SPBC1778.04 | spo6 | Spo4-Spo6 kinase complex regulatory subunit Spo6 |
| SPBC1861.06c | mug131 | UPF0300 family protein 4 |
| SPBC21D10.08c |  | Schizosaccharomyces specific protein |
| SPBC27.03 | meu25 | Schizosaccharomyces specific protein Meu25 |
| SPBC32H8.06 | mug93 | Paqosome core subunit, TPR repeat protein, human RPAP3 ortholog |
| SPBC428.07 | meu6 | pleckstrin homology domain protein Meu6 |
| SPBC4C3.08 | otg2 | alpha-1,3-galactosyltransferase Otg2 |
| SPBC56F2.03 | arp10 | dynactin complex actin-like protein Arp10 |
| SPBC8D2.19 | mde3 | serine/threonine protein kinase, meiotic, STKc MAK-like Mde3 |
| SPCC11E10.09c |  | alpha-amylase homolog |
| SPCC1235.13 | ght6 | plasma membrane glucose/fructose:proton symporter Ght6 |
| SPCC1259.14c | meu27 | UPF0300 family protein 5 |
| SPCC1682.12c | ubp16 | ubiquitin C-terminal hydrolase Ubp16 |
| SPCC188.12 | spn6 | meiotic (sporulation) septin Spn6 |
| SPCC1919.11 | mug137 | BAR adaptor protein, human endophilin A3-like |
| SPCC31H12.06 | mug111 | major facilitator family transmembrane transporter Mug111 |
| SPCC320.07c | mde7 | RNA-binding protein Mde7 |
| SPCC417.12 |  | carboxylesterase, type B family protein |
| **Late MUGs** | | |
| SPAC15E1.02c |  | DUF1761 transmembrane protein family, implicated in phosphate metabolism, stress response, sporulation |
| SPAC186.02c |  | hydroxyacid dehydrogenase, implicated in cellular detoxification |
| SPAC22G7.11c | cum1 | Con-6 family conserved fungal protein |
| SPAC23D3.05c |  | alcohol dehydrogenase pseudogene |
| SPAC2F7.06c | pol4 | DNA polymerase X family |
| SPAC3C7.02c | pil2 | meiotic eisosome BAR domain protein Pil2 |
| SPAC4F10.17 |  | plasma membrane and mitochondrial outer membrane, implicated in cell integrity signaling, conserved fungal protein |
| SPAC750.02c |  | transmembrane transporter |
| SPAC869.06c | hry1 | HHE domain cation binding protein, implicated in the repair of iron-sulfur clusters damaged by oxidative and nitrosative stress |
| SPAC869.07c | mel1 | alpha-galactosidase, melibiase |
| SPAC869.08 | pcm2 | protein-L-isoaspartate O-methyltransferase Pcm2 |
| SPAC869.09 |  | Con-6 family conserved fungal protein |
| SPAPB1A11.03 |  | FMN-dependent alpha-hydroxy acid dehydrogenase, probable lactate dehydrogenase |
| SPAPB8E5.10 | min8 | mitochondrial respiratory complex/ATP synthase complex assembly protein Mra1, meiosis specific splicing in fission yeast |
| SPBC685.03 |  | methyltransferase, sterol related |
| SPBC725.06c | ppk31 | serine/threonine protein kinase Ppk31 |
| SPBP4G3.03 | fub2 | PI31 proteasome regulator Fub2 |
| SPCC663.14c | trp663 | plasma membrane TRP-like ion channel |
| SPCC757.02c |  | dehydrogenase |
