# Supplementary material for: Nucleosome positioning shapes cryptic antisense transcription
Source: PLoS Genet. 2026 Mar 13;22(3):e1012078. doi: 10.1371/journal.pgen.1012078 (PMC13075793; doi:10.1371/journal.pgen.1012078)
Supplement: S3 Table — (DOCX) [file pgen.1012078.s015.docx]

**S3 Table**. Candidates for predicted interaction with the CHCT domain of Hrp3 with ipTM scores greater than 0.5.

| **Rank** | **Gene ID** | **NAME** | **IPTMavg** | **PTMavg** | **Description** |
| --- | --- | --- | --- | --- | --- |
| 1 | SPCC830.11c | Fap7 | 0.70198 | 0.6692 | Ribosome assembly chaperone for Rps14 |
| 2 | SPAC26A3.17c | Rmt2 | 0.6536 | 0.7382 | N-methyltransferase |
| 3 | SPBC713.05 | Wdr83 | 0.6412 | 0.6864 | Gpl1-Gih35-Wdr83 complex WD repeat subunit Wdr83 |
| 4 | SPAC22F3.05c | Alp41 | 0.5804 | 0.6466 | GTP-binding protein involved in beta-tubulin folding Alp41 |
| 5 | SPBC18E5.08 |  | 0.5618 | 0.6366 | N-acetyltransferase |
| 6 | SPBC651.09c | Prf1 | 0.5516 | 0.317 | RNA polymerase II associated Paf1 complex |
| 7 | SPBC685.04c | Aps2 | 0.521 | 0.5838 | AP-2 adaptor complex sigma subunit Aps2 |
| 8 | SPCC63.06 |  | 0.5188 | 0.7314 | WD repeat protein, human WDR89 family |
| 9 | SPAC1B3.12c | Rpb10 | 0.5146 | 0.513 | DNA-directed RNA polymerase I, II, and III subunit Rpb10 |
| 10 | SPCC1672.01 |  | 0.5046 | 0.7174 | histidinol-phosphatase |
| 11 | SPAC1782.08c | Rex3 | 0.5008 | 0.5926 | exonuclease Rex3 |
| 12 | SPCC1393.14 | Ten1 | 0.4982 | 0.5216 | telomere cap complex subunit Ten1 |
