# Supplementary material for: Nucleosome positioning shapes cryptic antisense transcription
Source: PLoS Genet. 2026 Mar 13;22(3):e1012078. doi: 10.1371/journal.pgen.1012078 (PMC13075793; doi:10.1371/journal.pgen.1012078)
Supplement: S4 Table — (DOCX) [file pgen.1012078.s016.docx]

**S4 Table.** *Schizosaccharomyces pombe* strains used in this study.

| Strain name | Genotype |
| --- | --- |
| YZH_001 | h+ ade6-M216 ura4-D18 leu1-32 |
| YZH_002 | h- ade6-M216 ura4-D18 leu1-32 |
| YZH_069 | h- ade6-M216 ura4-D18 leu1-32 hrp1-13xmyc-kanMX6 |
| YZH_070 | h- ade6-M216 ura4-D18 leu1-32 hrp3-13xmyc-kanMX6 |
| YZH_276 | h- ade6-M216 ura4-D18 leu1-32 ura4-D18∆::kanMX6-Pr_Spombe_Adh1-Ura4 |
| YZH_278 | h- ade6-M216 ura4-D18 leu1-32 ura4-D18∆::kanMX6-Pr_AT1TE70815-Sp_Ura4 |
| YJY_023 | h+ ade6-M216 ura4-D18 leu1-32 fft1Δ::hygMX |
| YJY_024 | h+ ade6-M216 ura4-D18 leu1-32 fft2Δ::kanMX |
| YJY_025 | h+ ade6-M216 ura4-D18 leu1-32 fft3Δ::kanMX |
| YJY_026 | h+ ade6-M216 ura4-D18 leu1-32 hrp1Δ::kanMX |
| YJY_027 | h+ ade6-M216 ura4-D18 leu1-32 hrp3Δ::kanMX |
| YJY_028 | h+ ade6-M216 ura4-D18 leu1-32 mit1Δ::kanMX |
| YJY_035 | h+ ade6-M216 ura4-D18 leu1-32 rrp1Δ::kanMX |
| YJY_036 | h+ ade6-M216 ura4-D18 leu1-32 rrp2Δ::kanMX |
| YJY_037 | h+ ade6-M216 ura4-D18 leu1-32 snf22Δ::hygMX |
| YJY_038 | h+ ade6-M216 ura4-D18 leu1-32 irc20Δ::kanMX |
| YJY_039 | h+ ade6-M216 ura4-D18 leu1-32 swr1Δ::kanMX |
| YJY_061 | h+ ade6-M216 ura4-D18 leu1-32 hrp1Δ::kanMX hrp3Δ |
| YJY_064 | h+ ade6-M216 ura4-D18 leu1-32 hrp1Δ::hrp1-CD(hrp3) hrp3Δ |
| YJY_065 | h+ ade6-M216 ura4-D18 leu1-32 hrp1Δ::kanMX hrp3Δ::hrp3-CD(hrp1) |
| YJY_070 | h+ ade6-M216 ura4-D18 leu1-32 hrp1Δ::hrp1-CT(hrp3) hrp3Δ |
| YJY_071 | h+ ade6-M216 ura4-D18 leu1-32 hrp1Δ::kanMX hrp3Δ::hrp3-CT(hrp1) |
| YJY_095 | h- ade6-M216 ura4-D18 leu1-32 ura4-D18∆::kanMX6-Pr_rap1(As)-Sp_Ura4 |
| YJY_096 | h- ade6-M216 ura4-D18 leu1-32 ura4-D18∆::kanMX6-Pr_atg9(As)-Sp_Ura4 |
| YJY_097 | h- ade6-M216 ura4-D18 leu1-32 ura4-D18∆::kanMX6-Pr_crt10(As)-Sp_Ura4 |
| YJY_098 | h- ade6-M216 ura4-D18 leu1-32 ura4-D18∆::kanMX6-Pr_spb70(As)-Sp_Ura4 |
| YJY_099 | h- ade6-M216 ura4-D18 leu1-32 ura4-D18∆::kanMX6-Pr_orc4(As)-Sp_Ura4 |
| YZH_276 | h- ade6-M216 ura4-D18 leu1-32 ura4-D18∆::kanMX6-Pr_Spombe_Adh1-Ura4 |
| YZH_278 | h- ade6-M216 ura4-D18 leu1-32 ura4-D18∆::kanMX6-Pr_AT1TE70815-Sp_Ura4 |
| YJY_122 | h+ ade6-M216 ura4-D18 leu1-32 prf1Δ |
| YJY_154 | h+ ade6-M216 ura4-D18 leu1-32 hrp1Δ::kanMX hrp3Δ prf1Δ |
| YJY_177 | h- ade6-M216 ura4-D18 leu1-32 hrp3-CHCT_R1(AlaScan)-13xmyc-kanMX |
| YJY_178 | h- ade6-M216 ura4-D18 leu1-32 hrp3-CHCT_R2(AlaScan)-13xmyc-kanMX |
| YJY_179 | h- ade6-M216 ura4-D18 leu1-32 hrp3-CHCT_R3(AlaScan)-13xmyc-kanMX |
| YJY_180 | h- ade6-M216 ura4-D18 leu1-32 hrp3-CHCT_R4(AlaScan)-13xmyc-kanMX |
| YJY_181 | h- ade6-M216 ura4-D18 leu1-32 hrp3-CHCT∆-13xmyc-kanMX |
