# Supplementary material for: Nucleosome positioning shapes cryptic antisense transcription
Source: PLoS Genet. 2026 Mar 13;22(3):e1012078. doi: 10.1371/journal.pgen.1012078 (PMC13075793; doi:10.1371/journal.pgen.1012078)
Supplement: Attachment — Submitted filename: 159914_1_rebuttal_3563368_t4ybp0.pdf [file pgen.1012078.s017.pdf]

Full Revision

Manuscript number: RC-2025-03122R
Corresponding author(s): Frédéric, Berger

1. General Statements

Dear Editor and Reviewers,

We sincerely thank you for your time and effort in reviewing our manuscript, titled "Nucleosome Positioning Shapes Cryptic Antisense Transcription." We appreciate the constructive feedback and valuable suggestions, which have helped us improve the quality and clarity of our work. Below, we provide a detailed response to each comment. For clarity, the reviewers' comments are reproduced in green, followed by our responses in plain text.

2. Point-by-point description of the revisions

Reviewer 1

This is an excellent study that leverages the chromatin biology of Schizosaccharomyces pombe to uncover the central role of CHD1-family remodelers in maintaining nucleosome organisation and suppressing cryptic transcription. The work is carefully executed. In short, the authors show that Hrp3 is the primary CHD1-family remodeler responsible for maintaining nucleosome organisation over gene bodies. This represses antisense transcription from cryptic promoters in gene bodies. They provide evidence that Hrp3 is repressed in meiosis to allow the induction of meiotic genes. They further identified that a conserved domain, the CHCT domain of Hrp3, is essential for its interaction with Prf1 (PAF complex subunit), which is critical for the chromatin organisation in gene bodies. This manuscript is of excellent quality and is an important contribution towards understanding how transcription initiation is repressed within gene bodies. I have small comments and suggestions for clarification.

We thank the reviewer for their thoughtful and generous assessment of our work. We appreciate the constructive suggestions and have addressed each point in the revised manuscript and response.

Comment 1: The study demonstrates that Hrp3 represses antisense transcription at meiotic genes, showing that Hrp3 is reduced in meiosis, which could facilitate the induction of meiotic genes. Is there a phenotype in the hrp3Δ or the hrp1Δ hrp3Δ mutant in relation to meiosis? E.g. do these strains enter meiosis uncontrolled?

We thank the reviewer for this insightful comment. We assessed meiotic phenotypes in *hrp3*Δ and *hrp1*Δ*hrp3*Δ (and, for completeness, *hrp1*Δ) by crossing each genotype on malt extract (ME) agar and

examined cells at 8 h and 24 h post–meiotic induction. For each genotype we prepared 6 independent replicates, randomized image sets, and had two blinded scorers classify cells into three categories: tetrads, conjugated pairs, and non-participants.

We did not observe the appearance of any cells undergoing meiosis in any of the four strains at 8 h post-meiotic induction, suggesting that the loss of *hrp1* or *hrp3* does not lead to uncontrolled meiosis. At 24 h post-meiotic induction (figure below), we observed that all mutants showed reduced tetrad formation and increased conjugated/non-participant cells relative to WT.

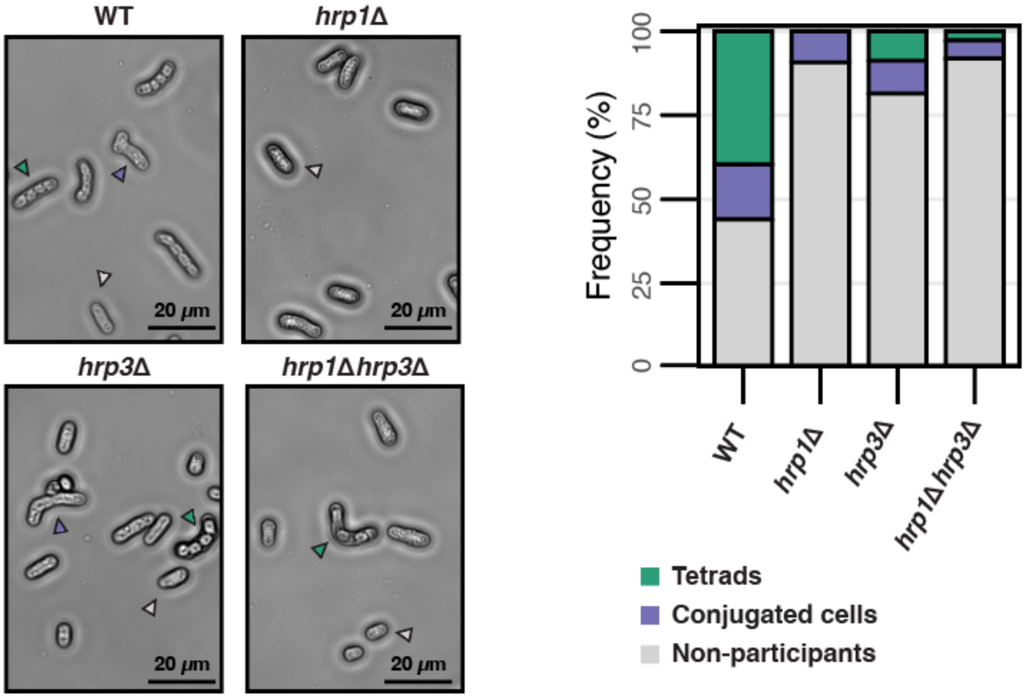

As a metric of meiotic progression efficiency, we calculated the ratio of tetrads to conjugated pairs (tetrad:conjugated) for each genotype. Conjugation marks successful cell fusion and entry into mating, whereas tetrad formation requires completion of both meiotic divisions and spore maturation. Thus, normalizing tetrads by conjugants controls for upstream mating efficiency and specifically reports the fraction of conjugants that successfully complete meiosis, and a decreased tetrad:conjugated ratio would indicate a defect during meiotic progression or sporulation downstream of conjugation. We show that this ratio is decreased in all mutants, indicating that progression through meiosis is also impaired.

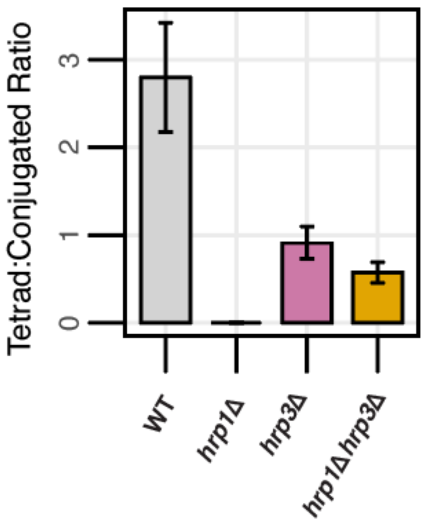

Our data show that loss of Hrp3 impairs normal meiotic progression, consistent with a model in which Hrp3 represses antisense transcription at convergently nested meiotic genes during vegetative growth so that these genes can be activated in a controlled program during meiosis. Notably, *hrp1*Δ also displayed a severe defect, however this is consistent with *hrp*1 being specifically upregulated during meiosis, suggesting that Hrp1 has acquired Hrp3-independent meiotic functions in fission yeast.

These findings were integrated as the new Figures **S6E-G**, and the results section as follows (lines 278–289):

'Upon mating and sporulation, *hrp3*Δ and *hrp1*Δ*hrp3*Δ showed reduced tetrad formation and accumulation of non-participant cells (**Fig. S6E, S6F**). As a proxy for efficient meiotic progression, we also calculated the ratio of tetrads to conjugated cells, and our results showed that meiosis progressed less efficiently in *hrp3*Δ and *hrp1*Δ*hrp3*Δ (**Fig. S6G**). Interestingly, *hrp1*Δ also showed a pronounced decrease in meiotic efficiency. Given that *hrp1* is upregulated during meiosis in comparison to *hrp3* (**Fig. 4F**), this phenotype may reflect an Hrp3-independent role for Hrp1 in meiotic progression. Taken together, these findings support a model in which Hrp3-dependent antisense repression prevents aberrant activation of convergently nested meiotic genes during vegetative growth, enabling their controlled induction during meiosis. This places Hrp3-mediated antisense control as a context-dependent layer coordinating expression of nested and meiotic genes in fission yeast.'

Comment 2: Figure 3C - ORC4 Locus TSS presentation. The presented data do not show a well-defined TSS on the sense strand. For reference, it would be useful to show that sense TSS is not altered between the different strains.

We thank the reviewer for this helpful suggestion. We acknowledge that the *orc4* gene does not exhibit a high level of expression, which results in a weak TSS peak in the PRO-seq data. To address this, we have included comparisons of browser snapshots with tracks from RNA-seq data in a new panel (**Fig. S3C**) to more clearly illustrate where transcription is initiating.

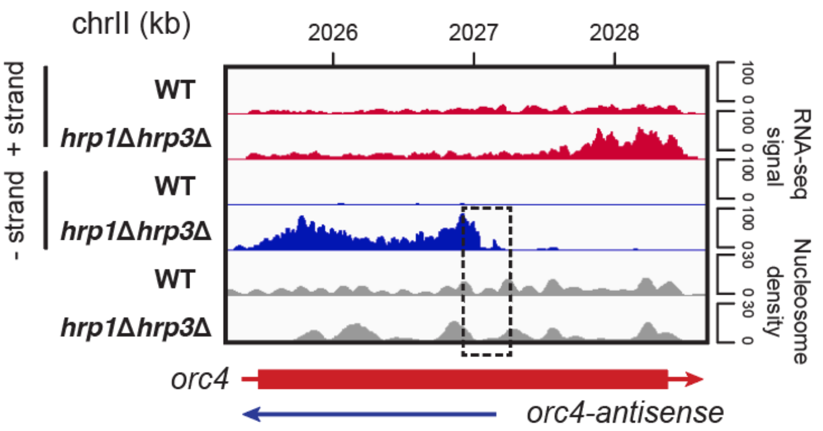

Additionally, to further support our findings, we have added genome browser tracks for another gene, *taf5*, located on the minus strand, which shows the same phenomenon of antisense initiation coinciding with nucleosome disruption, but with much more pronounced PRO-seq signal for at the sense TSS in both WT and *hrp1*Δ*hrp3*Δ (new **Fig. 3C** and **S3D**).

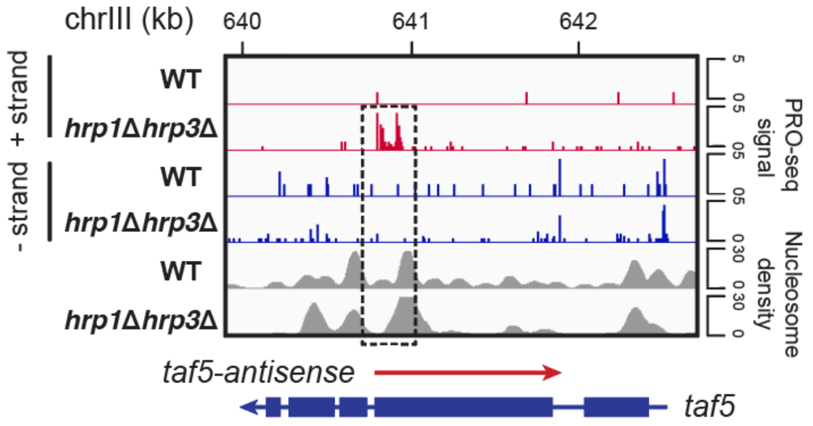

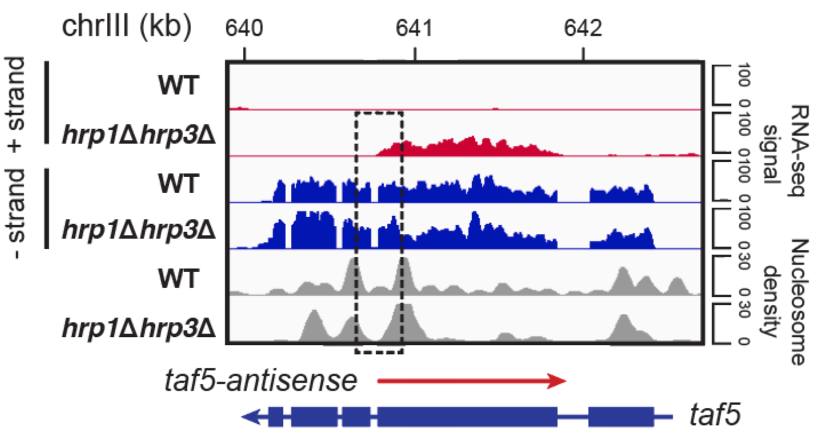

Additionally, a metaplot aligning the PRO-seq signal at the sense TSS also shows that both the magnitude and positioning of the sense TSS signal is relatively comparable between WT and all the other strains (**Fig. S3B**)

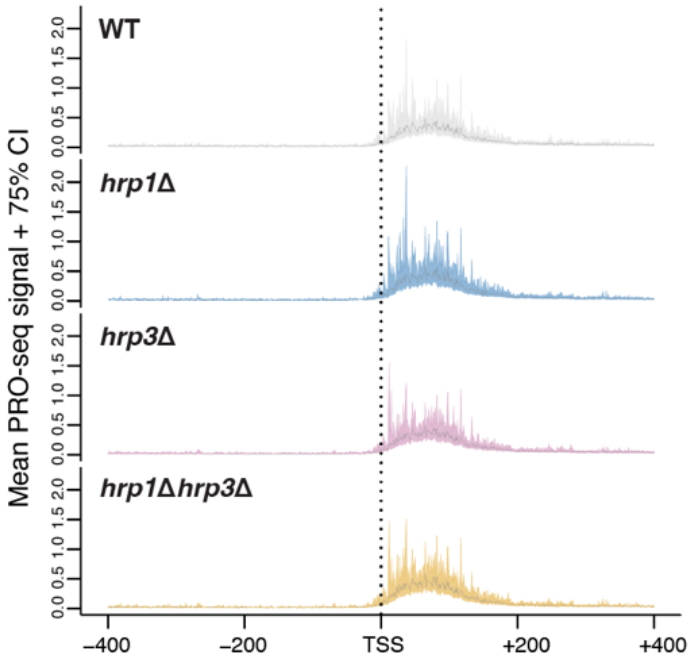

These additions provide a more comprehensive view of transcription initiation and ensure clarity for the reader. The updated supplementary figures have been included in the revised manuscript as Figures **3C**, **S3C** and **S3D**.

Comment 3: The study focuses on antisense cryptic transcription, which is relatively easy to measure by RNA-seq. Often, however, cryptic transcription can also occur in the sense direction in gene bodies. Do the

authors also find evidence of cryptic sense transcription in gene bodies (based on TSS-seq data)? This could be useful for completeness to report, as this could lead to aberrant protein-coding isoforms.

We appreciate the reviewer for their suggestion to assess cryptic sense initiation within gene bodies. Our study is centered on PRO-seq, which measures nascent transcription by engaged RNAPII. While PRO-seq reveals promoter-proximal pausing near annotated TSSs (which often occurs <200 bp from the actual TSS), it does not, by itself, resolve bona fide TSSs as cleanly as specialized TSS-mapping assays (e.g., CAGE-seq/Cap-seq). To address the question with our available data, we therefore performed two complementary analyses using orthogonal datasets: long-read Iso-Seq–informed annotations and strand-specific mRNA-seq.

First, we built an Iso-Seq–informed GTF annotation of cryptic sense transcripts. Then, we operated according to the rationale that if substantial sense cryptic initiation were occurring within gene bodies in the *hrp1*Δ*hrp3*Δ mutant vs WT, it should alter the intra-gene distribution of mRNA-seq reads (i.e., the profile shape) across the annotated sense isoforms. Such changes would appear as detectable shifts or distortions in mRNA-seq coverage profiles.

We then calculated the Pearson correlation between WT and *hrp1*Δ*hrp3*Δ sense coverage profiles. As a comparison, we repeated the correlation analysis for antisense coverage to directly compare effect sizes with those observed for sense transcription. We performed Wilcoxon rank-sum tests to compare the distributions of Pearson correlation coefficients between groups (sense transcription in WT – *hrp1*Δ*hrp3*Δ versus antisense transcription in WT – *hrp1*Δ*hrp3*Δ).

Our results showed that sense coverage profiles were highly concordant between WT and *hrp1*Δ*hrp3*Δ (median r close to 1.0), whereas antisense profiles showed substantially lower concordance with clear redistributions ($p < 1×10$^-16; median Pearson r for sense ≈ 0.94 vs antisense ≈ 0.53; Wilcoxon rank-biserial effect size = 0.532). These results argue against widespread emergence of new sense TSSs within gene bodies that would measurably distort sense profile shapes. This is now included as **Fig. S11**.

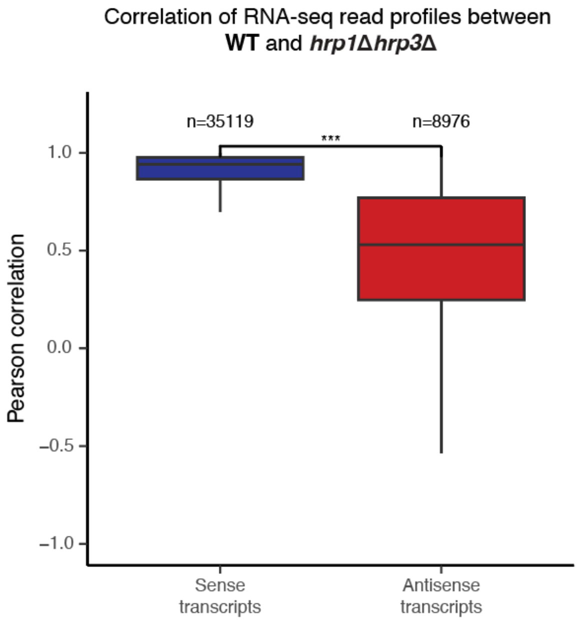

To corroborate these findings, we examined PRO-seq signal across annotated cryptic sense TSSs. In *hrp1*Δ, *hrp3*Δ and *hrp1*Δ*hrp3*Δ, we did not observe notable differences in the signal intensities compared to WT, or the emergence of metagene-level secondary peaks that would indicate pervasive novel sense initiation. By contrast, antisense metagenes showed robust increases consistent with our main findings (**Fig. 3A** in the manuscript).

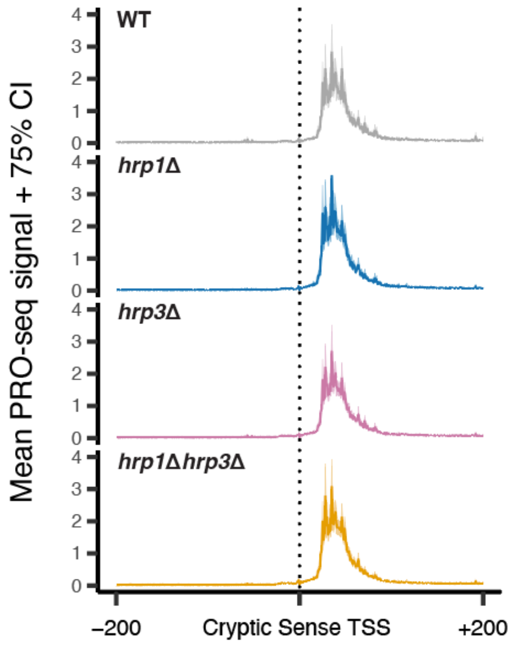

Within the constraints of our datasets, both the mRNA-seq profile analysis and the PRO-seq metagenes indicate that cryptic sense initiation, if present in the mutant, does not lead to production of cryptic sense transcripts at a level distinguishable from baseline sense transcription of the protein-coding gene—unlike the robust antisense cryptic initiation we can confidently report. Accordingly, we mention this but do not make claims about genome-wide cryptic sense initiation in this manuscript.

We have added the following text as **Supplementary Note 1**:

'To assess whether *hrp1*Δ*hrp3*Δ shows increased transcription of cryptic sense isoforms, we analyzed orthogonal datasets from long-read transcriptomics and mRNA-seq to assess intragenic sense initiation. We operated according to the rationale that if substantial sense cryptic initiation were occurring within gene bodies in the *hrp1*Δ*hrp3*Δ, it should alter the intra-gene distribution of reads (i.e., the profile shape) across affected loci compared to WT, even when total gene-level counts remain similar. Such changes would appear as detectable shifts or distortions in binned coverage profiles.
We generated a custom GTF of sense isoforms from Iso-Seq long reads (retaining transcripts overlapping curated PomBase genes) and, for WT and *hrp1*Δ*hrp3*Δ, merged strand-specific mRNA-seq replicates, computed sense-strand coverage along each Iso-Seq transcript (TSS→TES) in 50-bp bins, and correlated per-transcript binned profiles between genotypes; the same analysis was performed for antisense coverage and distributions were compared by Wilcoxon tests (**Fig. S11**). Sense coverage shapes were highly concordant between WT and *hrp1*Δ*hrp3*Δ (median Pearson $r \approx 0.95$), whereas antisense profiles showed strong redistribution (median $r \approx 0.53$; Wilcoxon $P < 1\times10$^−16; rank-biserial effect size = 0.663). These analyses argue against widespread, high-level cryptic sense initiation that would measurably distort sense gene-body profiles, in contrast to the pronounced antisense activation reported here; accordingly, we do not claim genome-wide cryptic sense initiation in this study.'

Comment 4: The manuscript alternates between "Prf1" (S. pombe) and "RTF1" (other eukaryotes). This is at times confusing. I recommend consistent use of gene nomenclature.

Response: We thank the reviewer for highlighting this inconsistency in gene nomenclature. To address this, we have revised the manuscript to use consistent terminology throughout. Specifically, we now use "Prf1/RTF1" when referring to the conserved protein across species and "Prf1" when specifically discussing the *S. pombe* homolog, ensuring clarity while maintaining accuracy. The changes have been implemented throughout the manuscript.

Comment 5: The authors show epistatic interaction for nucleosome spacing in Figure 7D for the prf1Δ and hrp1Δ hrp3Δ prf1Δ strains. It would be informative to have the hrp1Δ hrp3Δ data also included in Figure 7D, like in the other figure panels.

Response: We thank the reviewer for this insightful suggestion. To address this, we have now included the *hrp1Δhrp3Δ* data in Figure 7D (now **Figure 7F**), consistent with the other figure panels. This addition provides a more comprehensive comparison of the data. The updated figure has been included in the revised manuscript.

Full Revision

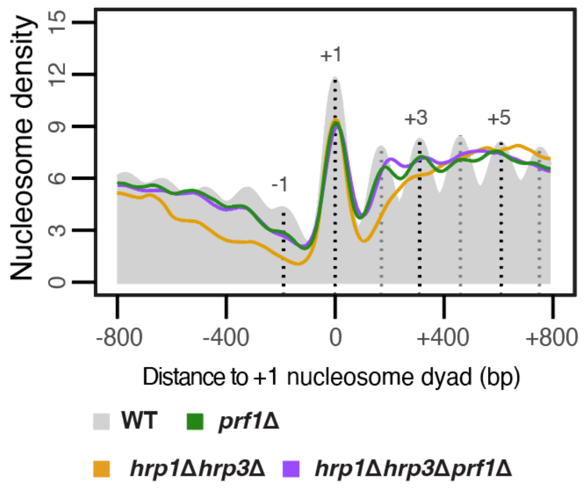

Reviewer 2

Kok et al. report on the role of the chromatin remodelers Hrp1 and Hrp3 in maintaining nucleosome positioning and preventing antisense transcription in Schizosaccharomyces pombe. As commented below, the main criticism of the manuscript is that the first half describes results that are very similar to those already reported by several other laboratories. Therefore, the main novel aspect of the work is the interaction between Hrp3 and the Prf1 subunit of the PAF complex.

We thank the reviewer for the thoughtful summary and for outlining the main points of emphasis. We appreciate the constructive feedback and address each of their comments in detail below, with corresponding revisions in the manuscript.

Comment 1: The articles of Hennig et al. (2012), Pointner et al. (2012) and Shim et al. (2012) are cited in the manuscript (line 119, Refs. 61-63) only as a confirmation of the minor effect of the absence of Hrp1 on nucleosome positioning and antisense expression. However, these three articles reached the same conclusion as Kok et al. that the absence of Hrp3 in S. pombe causes severe, genome-wide loss of nucleosome positioning and overexpression of antisense transcripts, whereas the absence of Hrp1 has a much weaker effect. These results were also discussed in a short review article (Touat-Todeschini et al. EMBO J. 2012. 31: 4371). Although Kok et al. analysed transcription at a higher resolution and mapped transcription initiation using Pro-Seq (Figures 1, 2 and 3), their results do not add much to what was already reported in these previous studies.

We thank the reviewer for highlighting important literature context for our study. We agree that Hennig et al. (2012), Pointner et al. (2012), and Shim et al. (2012) established that *hrp3Δ* causes a severe, genome-wide loss of nucleosome positioning with increased antisense transcription, whereas *hrp1Δ* has a comparatively modest effect, as also noted in Touat-Todeschini et al. (2012). We have revised the manuscript to acknowledge these studies more prominently and to frame our work as building upon their foundational observations. However, we also note that these studies did not establish the origins of these transcripts (cryptic promoters and TSSs), and the mechanism causing cryptic antisense transcription.

At the same time, our study extends these findings in several important ways:

1) Mapping where cryptic antisense transcription initiates: Using high-resolution RNA-seq, PRO-seq, and long-read RNA sequencing, we identify cryptic antisense TSSs genome-wide and show that they bear core promoter features (GC content, TATA class, TATA positional enrichment, and enriched TF motifs; see response to Reviewer 3, Major Comment 2). These analyses support bona fide promoter-like initiation within gene bodies. We also release a set of >5000 new antisense TSS annotations, together with motif calls as a community resource for *S. pombe*. This comprehensive accounting not only allowed us to perform more detailed mechanistic studies, but will also serve as an important resource to others studying the origins and impact of cryptic transcription.

2) Linking nucleosome phasing to repression of cryptic initiation: By jointly profiling nucleosome positioning and antisense TSSs at base-pair resolution, we find that Hrp3-established gene-body arrays occlude promoter-like sites and that phasing loss exposes these sites, thereby facilitating antisense initiation.

3) Delineating Hrp3 domain function and pathway relationships: We find that the Hrp3 CHCT domain is necessary for gene-body association and antisense repression, and our data support Prf1/RTF1 acting upstream of Hrp3:

   (i) Endogenous co-immunoprecipitation detects a Prf1–Hrp3 association (refer to our response to Comment 12).
   (ii) *prf1*Δ redistributes Hrp3 away from gene bodies and causes a stronger loss of H2BK119ub than *hrp3*Δ (refer to our response Reviewer 3, Comment 3).
   (iii) *prf1*Δ and *hrp1*Δ*hrp3*Δ*prf1*Δ display severe nucleosome dephasing and increased antisense comparable to *hrp1*Δ*hrp3*Δ.

   Taken together, these observations support a co-transcriptional mechanism in which Prf1 cooperates with Hrp3 to install gene-body H2Bub and nucleosome phasing that prevents transcription initiation at cryptic antisense promoters.

4) Systematic survey across remodeler families and H2A.Z exchange: We performed a genome-wide survey across major ATP-dependent remodeler families in *S. pombe*, benchmarking gene-body phasing and H2A.Z profiles around the TSS and TTS. This comparison corroborates the prominent phasing role for Hrp3 in our conditions and places it in context with other remodelers, while the H2A.Z analysis provides a reference framework for variant exchange relative to remodeler perturbations.

5) Hrp3-mediated antisense regulation of meiotic and nested genes: We uncover a link between Hrp3 and meiotic gene control. Several meiotic genes are upregulated in *hrp3*Δ during vegetative growth, many of which are convergently nested within larger host genes. These findings suggest that Hrp3-dependent antisense repression is co-opted to restrict meiotic gene expression outside meiosis. Consistent with this, *hrp3*Δ exhibits impaired meiotic progression (refer to our response to Comment 11).

Together, our work goes far beyond the established *hrp3*Δ phenotype: it (i) annotates cryptic antisense initiation genome-wide, (ii) quantitatively connects gene-body phasing to its repression, (iii) places Hrp3 within an elongation-coupled pathway via Prf1 and H2Bub, and (iv) provides high-quality genomic datasets for the community. We anticipate that these advances will complement prior studies and facilitate future work on chromatin and transcription.

Revisions in the manuscript

Introduction:

‘Prior work established that loss of the CHD-family remodeler Hrp3 disrupts nucleosome positioning genome-wide and induces pervasive antisense transcription, whereas its paralog Hrp1 has comparatively modest effects[63–66].’

Results:
‘By contrast, *hrp3*Δ exhibited a marked loss of nucleosome periodicity beyond the +2 position (**Fig. 1C**), consistent with prior reports implicating Hrp3 in gene-body phasing[63–66].’

‘Together, these genome-wide measurements of chromatin and RNA extend prior observations of antisense upregulation upon loss of CHD remodelers and demonstrate that CHD orthologs govern antisense transcription, with Hrp3 serving as the principal remodeler that suppresses antisense initiation.’

Discussion:
‘Our results build on earlier observations[63–66] that *hrp3*Δ disrupts gene-body phasing and elevates antisense transcription in *S. pombe* by defining where cryptic antisense transcripts initiate, identifying a Prf1-dependent recruitment pathway, and revealing a programmatic link to nested meiotic gene regulation.’

Comment 2: Several sites in the manuscript state that Hrp3 belongs to the SWI/SNF family of chromatin remodelers (for example, line 92). However, Hrp3 is a member of the CHD family, whose members have a very different structure and function (see, for example, Clapier et al. 2017. Nat Rev Mol Cell Biol 18: 407; Paliwal et al. 2024 TIGs 41:236).

We thank the reviewer for pointing out this discrepancy in our description of Hrp3. We agree that Hrp3 is a member of the CHD family of chromatin remodelers with distinct functions, and we have corrected this description throughout the manuscript.

Additionally, we have updated the introduction text to additionally cite the suggested references (Clapier et al. 2017 and Paliwal et al. 2024) to provide proper context.

Comment 3: The authors should indicate where the nucleosome remodelling activity of some of the proteins in Figure 1A like Irc20, Rrp1, Rrp2 and Mot1) has been reported.

We thank the reviewer for this suggestion. We have updated the introduction to indicate prior reports of any nucleosome remodeling or related functions for Fun30 (Fft1/Fft2/Fft3), Irc20, Rrp1/Rrp2 (Uls1) and Mot1, with the associated citations.

The updated text is as follows (lines 61-70):

‘Beyond the canonical CHD, ISWI, and INO80 families, several additional Swi2/Snf2 ATPase families have been implicated in chromatin remodeling–adjacent processes and transcriptional regulation. For example, Mot1/BTAF1 remodels TATA-binding protein (TBP)–DNA interactions to modulate promoter accessibility genome-wide[52,53], members of the FUN30 family have been reported to disassemble

nucleosomes during transcriptional elongation[54], and the Swi2/Snf2 proteins SHPRH/Irc20 and Uls1/HLTF carry RING-type ubiquitin ligase activity alongside the conserved ATPase domain and have been linked to remodeling events during DNA repair[55,56]. Nonetheless, among remodelers, robust nucleosome-spacing activity that establishes regular gene-body phasing is well supported primarily for the CHD, ISWI, and INO80 families.'

Comment 4: The analysis of nucleosome positioning by aggregating thousands of genes, such as those shown in Figure 1B, has low resolution and can only detect gross alterations affecting many genes. Nevertheless, several mutants, such as swr1Δ and rrp1Δ, also exhibit altered nucleosomal profiles in Figure 1B. In other cases, the occupancy of the first and second nucleosomes after the TSS is reduced relative to the wild type. Therefore, it cannot be concluded that "nucleosome arrays in wild type and most remodeller mutant cells were highly ordered and regular" (line 105).

We thank the reviewer for pointing this out. We agree that aggregate nucleosome profiles primarily reveal large, concerted changes and that some mutants (e.g., *swr1*Δ and *rrp1*Δ) show detectable deviations from WT in Figure 1B. We have revised the text to (i) acknowledge these mutant-specific differences and (ii) clarify that our main observation concerns the distinctive loss of gene-body phasing in *hrp3*Δ beyond the +2 nucleosome, which is not seen to the same extent in mutants lacking other remodelers.

The updated results section is as follows (lines 123-129):

'Meta-profiles of nucleosome positioning centered on the +1 nucleosome indicated that WT and several remodeler mutants retained broadly ordered promoter-proximal nucleosome arrays, although mutants such as *swr1*Δ and *rrp1*Δ exhibited alterations in +1/+2 nucleosome occupancy and overall amplitude (**Fig. 1B**). By contrast, *hrp3*Δ exhibited a marked loss of nucleosome periodicity beyond the +2 position (**Fig. 1C**), consistent with prior reports implicating Hrp3 in gene-body phasing[63–66]. This pattern was recapitulated in *hrp1*Δ*hrp3*Δ, whereas *hrp1*Δ alone showed minimal changes.'

Comment 5: Although it was previously reported that hrp3Δ mutants overexpress antisense transcripts (see point 1 above), it is unclear how this finding is represented in Figure 1D. Similarly, it not clear either why antisense transcription is undetectable in hrp1Δ relative to WT in Figure 1D, yet significantly higher than in WT in Figures 2B, 3A and 3B. Furthermore, sense transcription in the single and double mutants is comparable to WT in Figure 2A, yet much higher in Figure S3B.

We thank the reviewer for this suggestion and agree that the original display of Figure 1D was difficult to read. We have updated the figure to replace the previous visualization with boxplots. We believe this change substantially improves interpretability, as the plot now clearly show that the *hrp3*Δ mutant exhibits increased antisense transcription compared to the other remodeler mutants in the dataset.

Original:

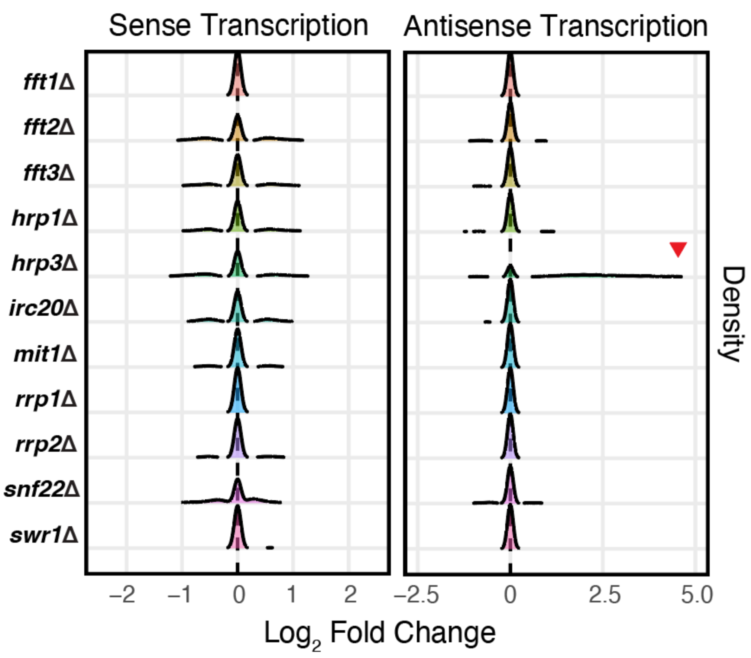

Updated:

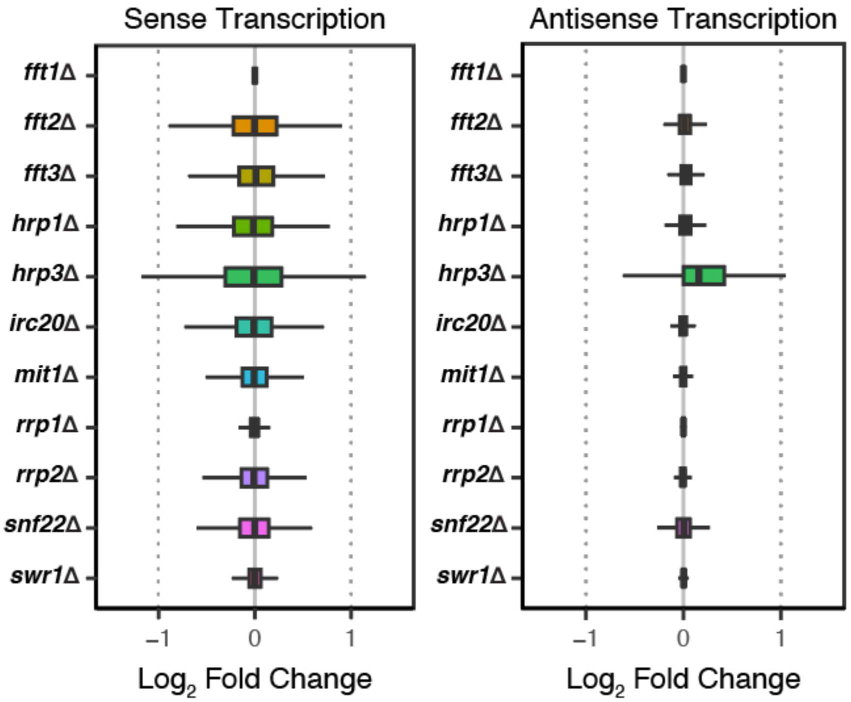

In addition, we appreciate the opportunity to clarify the inconsistencies pointed out with the antisense and sense signals across figures.

Regarding the differences between Figure 1D and 2B: Figure 1D summarizes gene-level log2 fold-changes from mRNA-seq for all remodeler mutants. For *hrp1*Δ, the median antisense shift is close to zero with a narrow IQR (i.e., the effect size is small at the transcriptome level) so the change appears minimal in this aggregate view. In Fig. 2B, the difference *hrp1*Δ vs WT reaches statistical significance because of high power across thousands of genes (meaning even small shifts will return statistical significance); however, the effect size remains small compared to *hrp3*Δ and *hrp1*Δ*hrp3*Δ, as reflected by the much smaller median shift and box height. In other words, *hrp1*Δ shows a statistically significant but minor antisense increase by mRNA-seq; *hrp3*Δ and *hrp1*Δ*hrp3*Δ show much larger antisense increases.

Regarding Figures 3A and 3B: Both figures use PRO-seq, which measures nascent transcription and is more sensitive to initiation-proximal changes than steady-state mRNA-seq. Consequently, the antisense increase for *hrp1*Δ appears larger in the PRO-seq TSS-proximal metrics than in gene-level mRNA-seq summaries, which integrate over processing, stability, and termination. This assay difference can explain why *hrp1*Δ shows minimal shifts in Fig. 1D, small but significant changes in Fig. 2B, and a clearer TSS-proximal increase in Fig. 3A and 3B, without contradicting the stronger *hrp3*-dependent phenotype.

Regarding Figures 2A and S3B: We realized that in Fig. 3B the WT signal (light gray) blended into the white background, making the WT signal appear lower than it is. To avoid this misperception, we have updated the figure to use a darker gray for WT. In addition, the shaded area for each strain represents the 75% confidence interval around the mean PRO-seq metagene, which may have visually overstated differences between strains. We have darkened and thickened the mean trace; the mean profiles show minimal differences across strains, consistent with Fig. 2A.

Original:

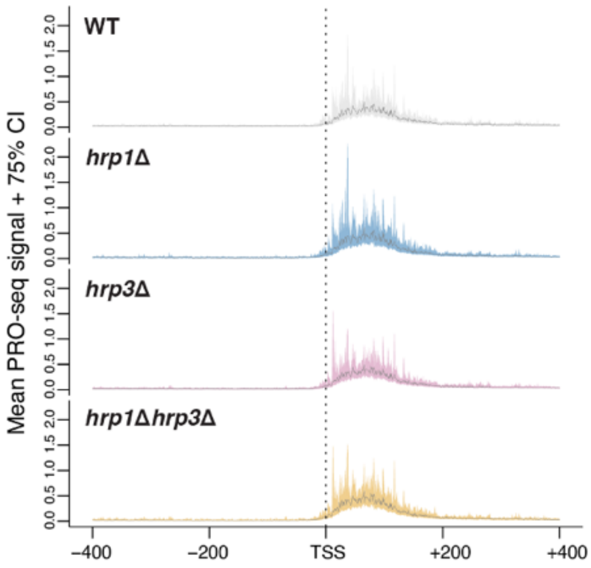

Updated:

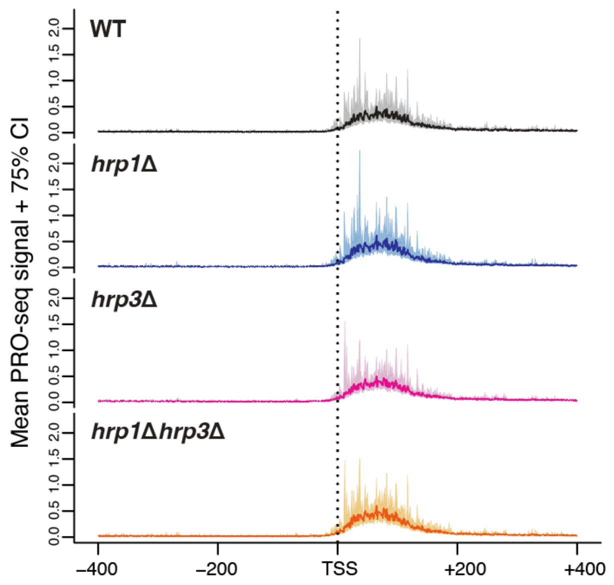

For consistency and clarity, we applied the same visual adjustments to Fig. 3A as well.

Comment 6: Figure S3C claims that antisense transcription is higher in genes with greater nucleosome disruption in the double mutant hrp1Δhrp3Δ. However, without a quantitative analysis, it is difficult to discern any significant differences in the degree of disruption across the four quartiles of antisense expression.

We appreciate the reviewer for this comment and agree that the original panel (Fig. S3C) did not provide a sufficiently quantitative basis for the stated conclusion. After re-examining the data, we revised both the analysis and the interpretation.

Revised quantification: We computed the 152 bp phasing component using a fast fourier transform (FFT) on gene-body MNase profiles (300–1200 bp from the TSS) for genes binned by antisense expression quartiles defined in *hrp1*Δ *hrp3*Δ. 152 bp was chosen for the phasing component as the nucleosome repeat length in *S. pombe* has previously been reported to correspond to this distance in several studies. Our results show that the nucleosome phasing amplitude increases across quartiles in WT, whereas it remains low and only modestly varying in the *hrp1*Δ *hrp3*Δ mutant.

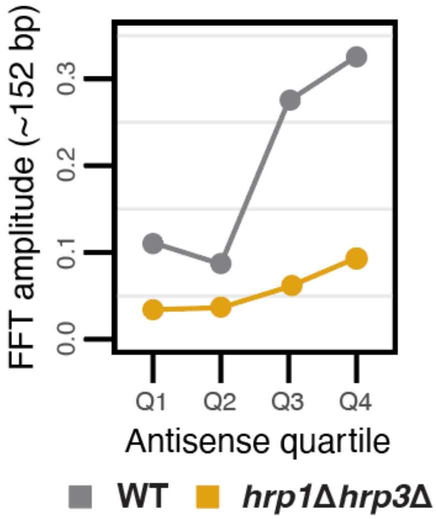

Revised interpretation: Rather than concluding that antisense transcription is higher in genes with greater disruption in the double mutant, we now conclude that genes that display higher antisense expression in *hrp1*Δ*hrp3*Δ are precisely those that exhibit stronger, well-phased arrays in WT. This pattern indicates that these genes are particularly phasing-dependent in WT and become de-phased and antisense-prone when remodeler function is compromised.

The new figure is included as **Figure S3F**, and we updated the text in the results section (lines 177-182):

'When we stratified genes by *hrp1*Δ*hrp3*Δ antisense expression, the WT showed increasing gene-body phasing with higher antisense quartiles, whereas *hrp1*Δ*hrp3*Δ exhibited uniformly low amplitudes of nucleosome phasing (**Fig. S3E, S3F**). This suggests that genes that exhibit high antisense expression in *hrp1*Δ*hrp*3Δ are those that normally depend on robust gene-body phasing, linking remodeler-dependent phasing to suppression of cryptic antisense transcription.'

Comment 7: Figures 3D and S4C show that the TSS of antisense transcription colocalizes with a region resistant to MNase that is at least 300 bp wide. This size does not correspond to that occupied by a nucleosome and contrasts with the expected size of the four nucleosome peaks downstream from it.

We thank the reviewer for this observation. We agree that the MNase-resistant region centered on antisense TSSs appears wider (~300 bp) than the ~150 bp expected for a single nucleosome when viewed as a meta-profile, in contrast to the regularly spaced peaks downstream.

Our interpretation is that this broadening reflects positional heterogeneity of a single (+1-like) nucleosome relative to the antisense TSS—across genes (and likely across cells)—rather than an unusually large particle. To demonstrate across-gene variability, we re-visualized the data of the WT strain as a heatmap aligned at antisense TSSs. The heatmap shows a narrow MNase-protected peak per gene whose position

shifts among genes, producing a smeared band in the aggregate (heatmap below). Downstream nucleosomes retain ~150 bp periodicity, consistent with canonical spacing.

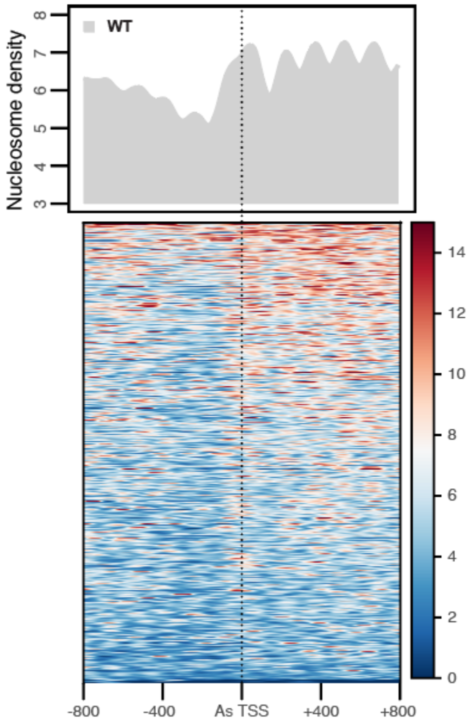

Despite the heterogeneity, a zoomed view highlights two favored positions of this nucleosome (just upstream or just downstream of the antisense TSS), which explains the broad peak with a shoulder in the meta-profile.

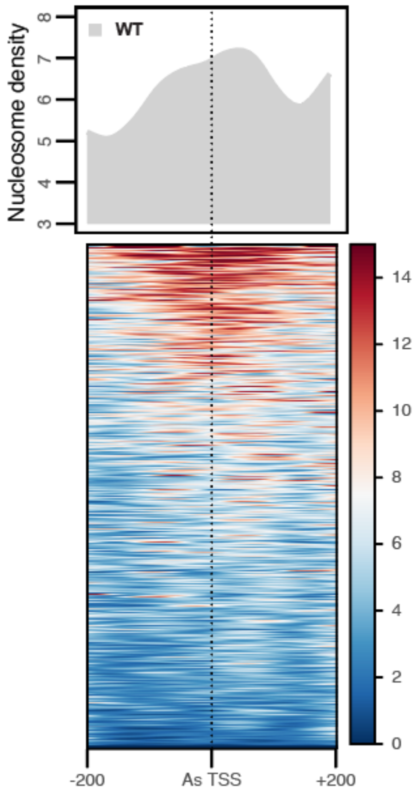

We have added the zoomed view as a new supplementary figure (**Figure S3G**) and updated the text in the results (lines 184-189) to highlight this observation in more detail:

'In all strains, the aggregate meta-profiles showed an apparently broad MNase-resistant feature at the As-TSS (**Fig. 3D**). Heatmap visualization indicated that this broadening reflected positional heterogeneity of the nucleosome immediately adjacent to the As-TSS across genes, rather than an atypically large particle (**Fig. S3G**). By contrast, downstream nucleosomes retained canonical spacing, with peak-to-peak distances of ~150 bp.'

Comment 8: In relation to the previous point, Figure S4C (bottom) shows that the centre of the region above the TSS is slightly displaced in the three mutants. This displacement corresponds to an increase in the G+C content of approximately 1.5% (Figure S4C top), equivalent to an increase of less than 2.5 Gs and Cs every 150 bp of nucleosomal DNA. Without some cause and effect experiments, it is difficult to attribute a functional significance to such a tiny difference. How repetitive is this difference in biological replicates?

We thank the reviewer for this careful observation. We re-examined the apparent displacement in Fig. S4C and evaluated its reproducibility across biological replicates (figure below). The shift was not reproducible, and the associated GC-content difference was very small (~1–2%), so we do not ascribe functional significance to it. Consequently, we have removed the claim and revised the figure and text accordingly.

Full Revision

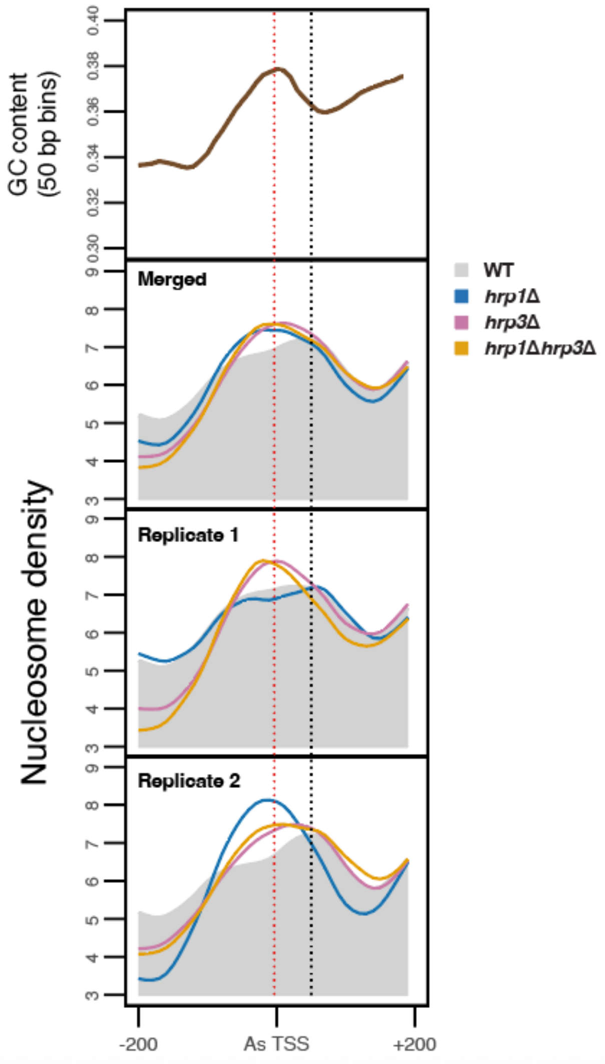

While revisiting these analyses, we also discovered that the original Fig. 3D had been generated with an outdated antisense annotation file; we corrected this error and regenerated the figure with the current annotation. The updated meta-profiles differ only slightly at the nucleosome over the antisense TSS in the mutants and do not alter any conclusions of the manuscript.

Original:

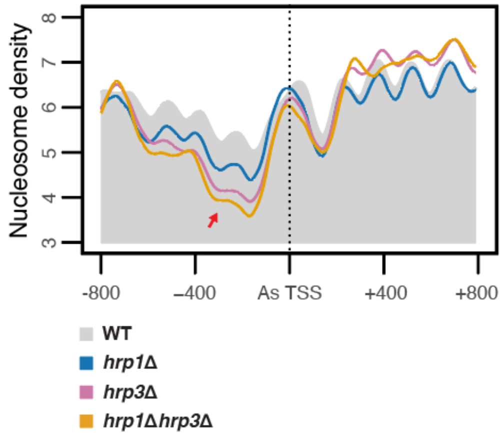

Updated:

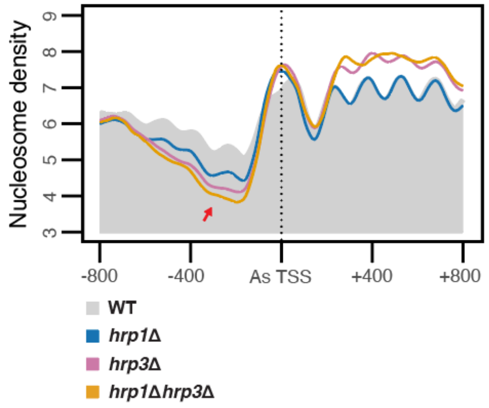

Comment 9: The authors should also explain how the position of the dyads was estimated in the double mutant hrp1Δhrp3Δ in Figure S4B. The severe loss of nucleosomal positioning suggests that the dyads occupy different positions in different cells within the same population. While most of the remaining figures show data for the three mutants, this figure shows results for the double hrp1Δhrp3Δ mutant only.

We thank the reviewer for this comment. Dyad positions in *hrp1Δhrp3Δ* were estimated using the same pipeline applied to all genotypes. We updated the methods to include the following (lines 994-998):

'For the calling of nucleosome dyad positions, we used the Numap package from Choi, Lyons et al. (2020) (https://github.com/orphancode/NuMap, https://github.com/dblyons/MNase_seq) with the call_dyads function on default parameters. For each dyad, ±50 bp windows (100 bp) were extracted with bedtools (v2.27.1) getfasta. GC% was computed as (G+C)/(A+C+G+T) × 100 using Python (v3.6.6). Distributions and statistical tests were plotted in R (v4.3.1).'

In response to the request to show all mutants, we now provide GC-content distributions for dyad-centered windows for WT, *hrp1Δ*, *hrp3Δ*, and *hrp1Δhrp3Δ* in the updated panel (**Fig. S4B**), replacing the prior

version that showed only the double mutant. The same calling and processing pipeline was used across strains, and the figure reports mean GC values together with Kolmogorov–Smirnov statistics comparing the GC-content distribution of each mutant versus WT. These additions address the request for all mutants without altering our conclusions.

Original:

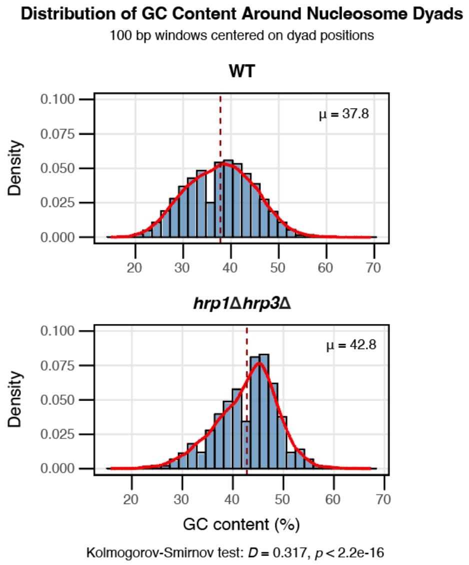

Updated:

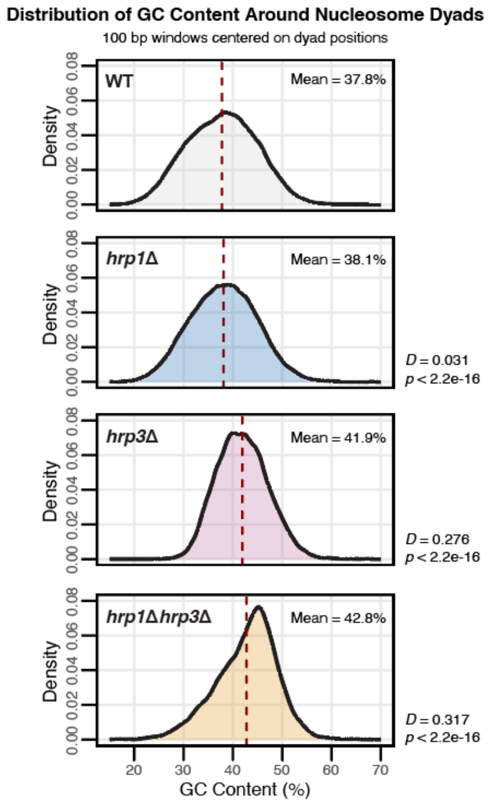

The pronounced loss of positioning in *hrp1*Δ*hrp3*Δ likely reflects increased positional heterogeneity of nucleosomes across cells. Importantly, Numap calls dyads on a per-locus basis rather than from meta-aggregated profiles; thus, heterogeneity does not invalidate dyad calling, but appears as lower-amplitude/broader local peaks and, accordingly, fewer or lower-confidence calls at affected loci. Even so, dyads in *hrp1*Δ*hrp3*Δ retain a stronger bias toward higher-GC sequence compared with WT, consistent with our genome-wide distributions. An illustrative locus (*orc4*; see below) shows elevated antisense transcription in the double mutant accompanied by broader MNase peaks whose positions preferentially overlap higher-GC regions.

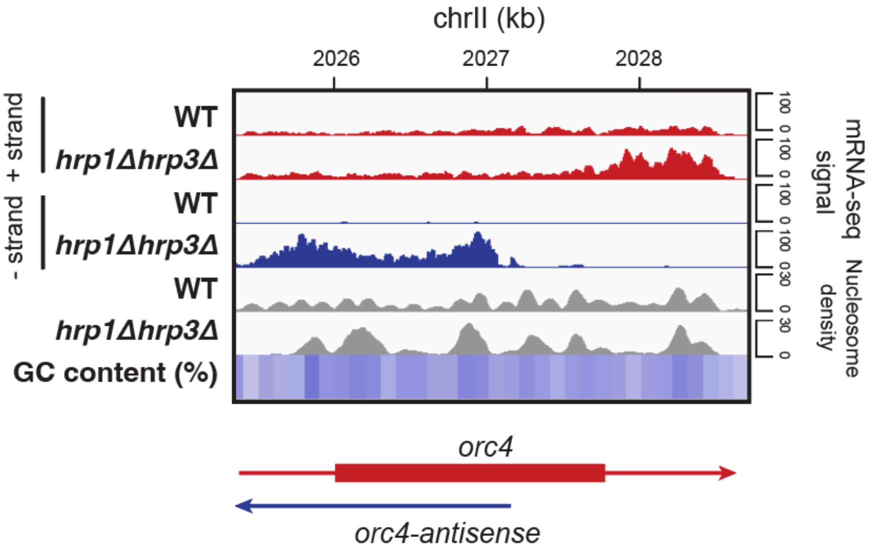

Comment 10: Figures 3G and 3H show the analysis of the promoter activity of some regions upstream from antisense transcripts, achieved by replacing the endogenous ura4 gene promoter with these regions. This analysis lacks negative controls showing the level of transcription in the recipient strain following the removal of the endogenous ura4 promoter and its replacement for genomic regions not associated with the initiation of antisense transcription in the mutants. Furthermore, transcription should be measured by quantitative PCR of the ura4 mRNA rather than by the more indirect method of measuring OD600 in 384-well plates (line 708).

We thank the reviewer for this helpful suggestion. We repeated the assay using RT-qPCR of *ura4* mRNA and added an explicit negative control strain in which the endogenous *ura4* promoter was deleted without replacement. Specifically, we measured *ura4* transcript levels (normalized to *act1*) in strains where the *ura4* promoter was replaced by 500-bp regions upstream of antisense TSSs (*crt10*, *atg9*, *orc4*, *spb70*, *rap1*) or by the *adh1* promoter (positive control) and compared these to the promoter-deleted negative control.

The RT-qPCR results corroborate the OD600-based measurements: regions that supported growth also drove significantly higher *ura4* mRNA than the negative control (one-sample t-test of log2FC against 0).

Growth-based assay:

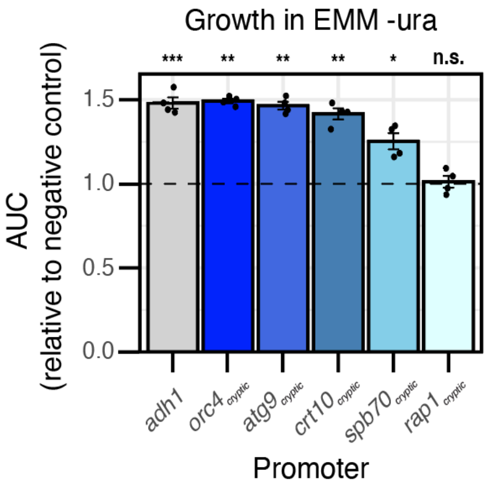

qPCR-based assay:

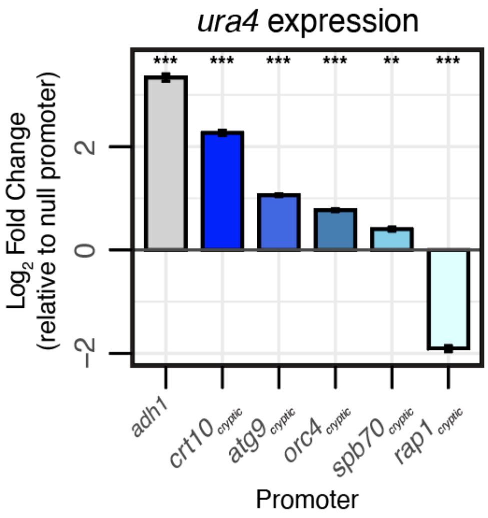

We note that expression in the growth-based assay appears to plateau for the strongest constructs, consistent with *ura4* transcription no longer being the limiting factor for growth in the assay. Interestingly, the *rap*1 antisense region drove lower *ura*4 mRNA than the negative control, suggesting that in this context it may harbor repressive activity that we weren't able to capture in our growth-based assay. Given the superior dynamic range of the RT-qPCR assay, we have replaced the growth-based panel with RT-qPCR as the primary readout (new **Figure 3I**), retained the negative control explicitly in the figure, and revised the text accordingly. These additions address the request for a proper negative control and a direct transcriptional measurement of the *ura4* locus to support antisense promoter activity.

The updated in the results section is as follows (lines 238–243):

‘To test whether antisense-upstream regions can function as promoters, we replaced the endogenous *ura4* promoter with 500-bp regions upstream of the antisense TSSs from five loci (**Fig. 3H**) and quantified *ura4* mRNA by RT-qPCR (normalized to *act1*), including a promoter-deleted strain as a negative control and the *adh1* promoter as a positive control. Apart from *rap1*, four of the five regions (*crt10*, *atg9*, *orc4*, *spb70*) produced significantly higher *ura4* mRNA than the negative control, supporting their intrinsic promoter activity (**Fig. 3I**).’

For additional evidence that these antisense promoters share core features with typical protein-coding promoters in fission yeast, please see our response to Reviewer 3, Major Comment 2.

Comment 11: Figure F4 suggests that Hrp3 may regulate the expression of genes specific to meiosis by showing an anticorrelation between the expression levels of Hrp3 and a selection of genes that are upregulated during meiosis (MUGs) 5 hours after the onset of meiosis. While this is an interesting possibility, it will remain speculative until it is demonstrated that the level of Hrp3 protein is reduced at the same stage of meiosis, and that MUG overexpression is associated with reduced nucleosomal occupancy adjacent to their TSS at that stage.

We appreciate this thoughtful suggestion. Directly demonstrating reduced Hrp3 protein at the 5 h meiotic timepoint and concomitant nucleosome changes at MUG promoters would require a dedicated and synchronized meiotic time-course with protein quantification and MNase profiling, which is outside the scope of the present study.
These data were partially presented in the context of Reviewer 1, Comment 1 above, and are reproduced below.

To probe functional relevance, however, we assessed meiotic phenotypes in *hrp3*Δ, *hrp1*Δ, and *hrp1*Δ*hrp3*Δ by mating each genotype on malt extract (ME) agar and scoring cells at 8 h and 24 h post-induction. Across 6 replicates per genotype, randomized images were scored by two independent researchers as tetrads, conjugated pairs, or non-participants. By 24 h post-meiotic induction (figure below), we observed that all mutants showed reduced tetrad formation and increased conjugated/non-participant cells relative to WT.

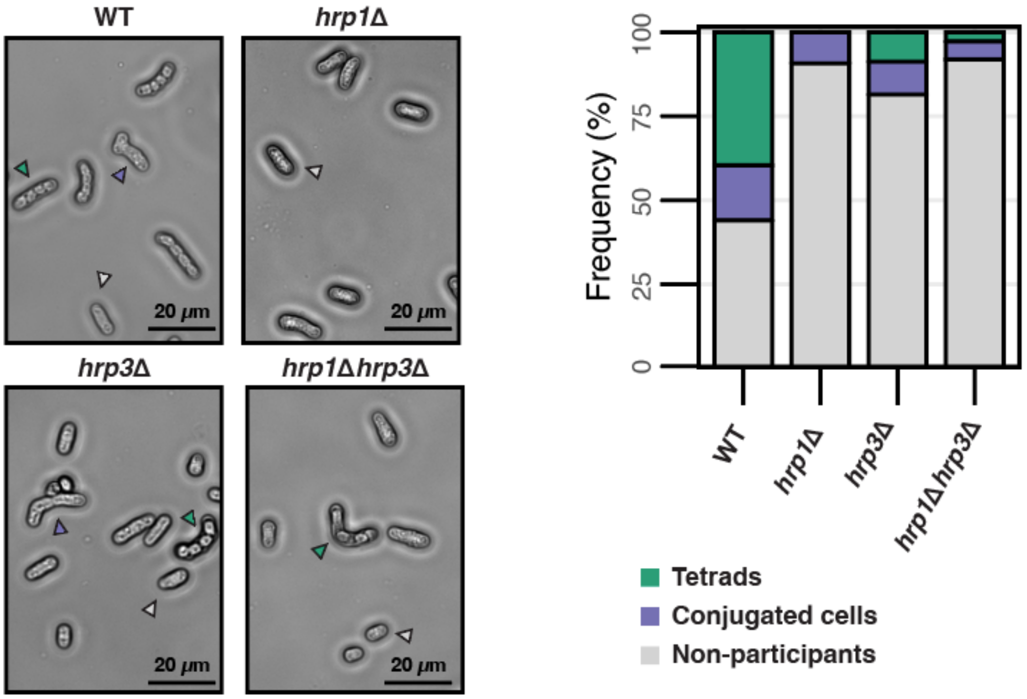

As a metric of meiotic progression efficiency, we calculated the ratio of tetrads to conjugated pairs (tetrad:conjugated) for each genotype. Conjugation marks successful cell fusion and entry into mating, whereas tetrad formation requires completion of both meiotic divisions and spore maturation. Thus, normalizing tetrads by conjugants controls for upstream mating efficiency and specifically reports the fraction of conjugants that successfully complete meiosis, and a decreased tetrad:conjugated ratio would indicate a defect during meiotic progression or sporulation downstream of conjugation. We show that this ratio is decreased in all mutants, indicating that progression through meiosis is also impaired.

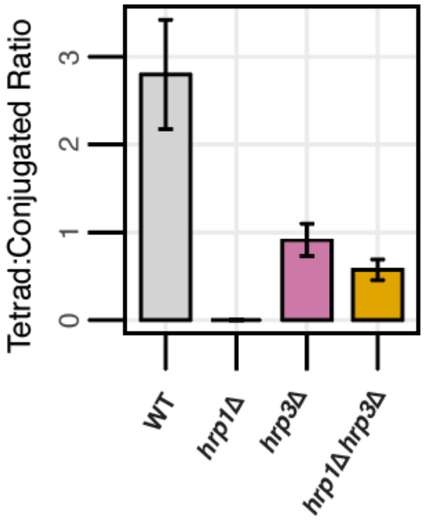

Our data show that loss of Hrp3 impairs normal meiotic progression, consistent with a model in which Hrp3 represses antisense transcription at convergently nested meiotic genes during vegetative growth so that these genes can be activated in a controlled program during meiosis. Notably, *hrp1*Δ also displayed a severe defect, however this is consistent with *hrp*1 being specifically upregulated during meiosis, suggesting that Hrp1 has acquired Hrp3-independent meiotic functions in fission yeast. While we agree that time-resolved Hrp3 protein measurements and nucleosome profiling at MUG promoters would be valuable, we view them as an important direction for future work rather than a requirement for the current study's conclusions.

These findings were integrated into the results section as well as the new figure panels **S6E-G**:

'To determine whether the Hrp3-dependent meiotic repression resulted in any defects during meiosis, we mated WT, *hrp1*Δ, *hrp3*Δ, and *hrp1*Δ*hrp3*Δ strains and examined tetrad formation 24 h after induction on ME agar. Relative to WT, all mutants showed reduced tetrad formation and accumulation of non-participant cells (**Fig. S6E, S6F**). As a proxy for efficient meiotic progression, we also calculated the ratio of tetrads to conjugated cells, and our results showed that meiosis progressed less efficiently in *hrp3*Δ and *hrp1*Δ *hrp3*Δ (**Fig. S6G**). Interestingly, *hrp1*Δ also showed a pronounced decrease in meiotic efficiency. Given that *hrp1* is upregulated during meiosis in comparison to *hrp3* (**Fig. 4C**), this phenotype may reflect an Hrp3-independent role for Hrp1 in meiotic progression. Taken together, these findings are consistent with a model in which Hrp3 represses antisense transcription at convergently nested meiotic genes during vegetative growth so that these genes can be activated in a controlled program during meiosis in fission yeast.'

Comment 12: The experiments in Figures 5 and 6, which describe the interaction between the Hpr3-specific CHCT domain and the Prf1 protein, are interesting and represent the main element of novelty of the manuscript. However, this interaction in figure 6D and 6E should be confirmed in vivo.

We agree that *in vivo* validation is important and have performed co-immunoprecipitation in cells expressing Prf1-FLAG and Hrp3-Myc from their endogenous loci. Whole-cell extracts were immunoprecipitated with anti-FLAG (M2) beads and probed by immunoblotting. Hrp3-Myc co-precipitated with Prf1-FLAG in two independent biological replicates (see below). The signal is modest but reproducible across replicates, consistent with a specific yet transient interaction *in vivo*.

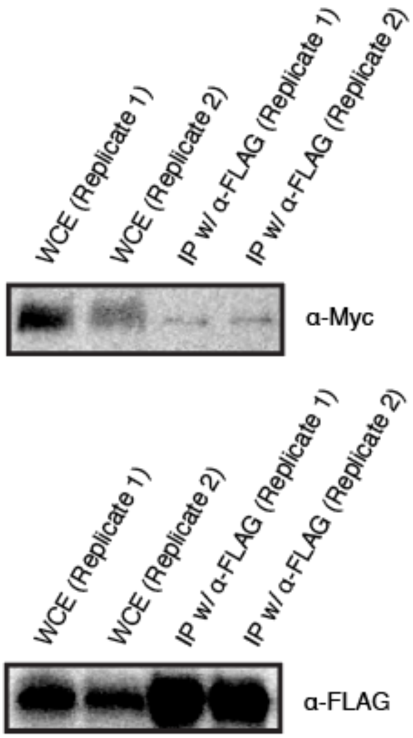

Together with our *in vitro* results, the endogenous co-IP supports a specific Hrp3–Prf1 interaction *in vivo.*

We have added our results as a new figure panel and updated the text in the results section as follows (lines 360-363):

‘To validate the predicted interaction in cells, we first performed endogenous co-immunoprecipitation from strains expressing Prf1-FLAG and Hrp3-Myc. Anti-FLAG IP recovered a modest but reproducible amount of Hrp3-Myc (**Fig. S8I**), consistent with a specific but likely transient association *in vivo*.’

Comment 13: Kok et al. indicate that the triple prf1Δ hrp1Δ hrp3Δ mutant exhibits stronger growth defects than the single prf1Δ mutant. However, Figure S9F shows that no growth is detectable in the single prf1Δ mutant, a phenotype that cannot be exacerbated in the triple mutant. Perhaps the use of a prf1 mutant showing a less severe phenotype might help.

We thank the reviewer for pointing this out. The *prf1*Δ strain indeed grows very poorly on plates, and we recognize that this can be difficult to visualize.

To quantify growth more directly, we measured endpoint OD600 in liquid culture (YES, 32°C, 15 h; n = 3 biological replicates; see below). A Mann–Whitney U test showed that *prf1*Δ*hrp1*Δ*hrp3*Δ had significantly reduced growth compared to *prf1*Δ (p = 4.95e-2).

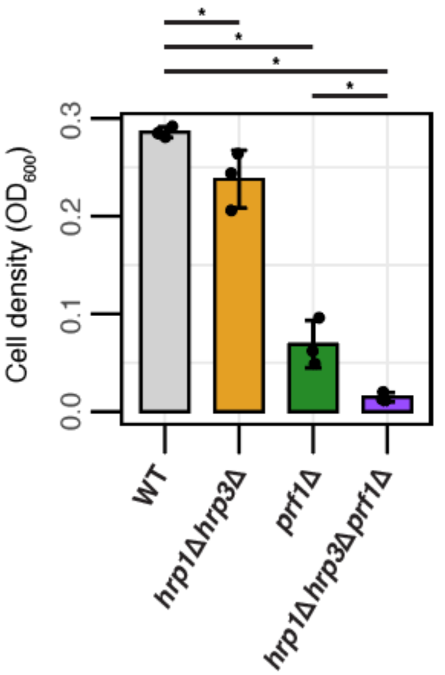

We have replaced the original figure with the liquid-culture growth comparison (**Fig. S10H**). These additions are consistent with the conclusion that the triple mutant exhibits a stronger growth defect under our conditions.

Reviewer 3

In the manuscript "Nucleosome positioning shapes cryptic antisense transcription", Kok and colleagues perform a characterization of nucleosome remodeling factors in S. pombe by assaying the impact of their deletion on antisense transcription and nucleosome organization. They find that deletion of Hrp3 leads to up-regulation of antisense RNA transcripts as well as disruption of phased nucleosomes in gene bodies. The authors then establish a catalogue of antisense transcripts in S. pombe using long read RNA sequencing, which they use to analyze the relationship between nucleosome positioning and antisense transcription. Through this analysis, they associate nucleosome positioning with the initiation of antisense transcription and conclude that nucleosome positioning within gene bodies represses cryptic antisense transcription. They further support this observation by showing that the up-regulated genes in the Hrp3 knock-out are enriched for genes usually expressed in meiosis, which in S. pombe often occur as nested transcripts in reverse orientation. Using growth assays under various stress conditions, the authors narrow down the domain responsible for the phenotype to the C-terminal CHCT domain. To address how Hrp3 gains specificity, they perform an in-silico interaction prediction screen to identify Prf1 as a putative interactor of the CHCT domain. Using recombinant expression in bacteria followed by pulldowns from lysates, they confirm the interaction and introduce point mutants that abolish the interaction. The authors then link the interaction with Prf1 to transcriptional elongation, where they observe a correlation between Hrp3 presence and chromatin marks of transcription elongation, especially H2BK119ub, which is also reduced in the Hrp3 knockout. They further demonstrate that both gene body nucleosome phasing and antisense transcription are similarly affected in the prf1 knockout as well as the hrp1-hrp3-prf1 triple knock-out cells, which indicates that they affect the same pathway.

We thank the reviewer for the clear and constructive summary of our work. We appreciate the thoughtful assessment and address their specific comments point-by-point below, with corresponding revisions to the manuscript.

Major Comment 1: The authors use the terms "Prf1" and "Paf1 complex" interchangeably multiple times in the manuscript (eg. Line 296). However, the experimental data presented only demonstrate a connection between Prf1 and Hrp3. Furthermore, published literature establishes that Prf1 and Paf1 represent distinct entities in S. pombe (Mbogning et al., 2013, PLoS Genetics 9(3): e1004029). The authors should clarify this distinction and use consistent, accurate terminology throughout the text.
Reference: Mbogning, J., et al. (2013). The PAF Complex and Prf1/Rtf1 Delineate Distinct Cdk9-Dependent Pathways Regulating Transcription Elongation in Fission Yeast. PLoS Genetics, 9(3), e1004029. https://doi.org/10.1371/journal.pgen.1004029

We thank the reviewer for pointing out this important distinction and for highlighting the need for consistent terminology. To address this, we have carefully revised the manuscript to consistently refer to Prf1 as a "transcriptional regulator" rather than a "Paf1 complex subunit".

Additionally, we have clarified in the results section that Prf1 is only biochemically part of the Paf1 complex in budding yeast, and this does not hold in *S. pombe* and metazoans. We have also cited the literature (Mbogning et al., 2013) to provide further context and ensure accuracy.

The additional text in the results is as follows (lines 348-350):

'Notably, while Prf1/Rtf1 is a stable component of the elongation-associated Paf1 complex in budding yeast, it is biochemically distinct in metazoans and in *S. pombe*.'

Major Comment 2: The authors demonstrate that Hrp3 limits antisense promoter usage; however, the analysis lacks characterization of sequence composition, promoter classes (TATA-box versus TATA-less), or identification of enriched transcription factor motifs near these sites. A more thorough bioinformatic analysis would strengthen the paper and potentially reveal interesting biology, as the effect may be specific to certain transcription factors or promoter architectures.

We thank the reviewer for this valuable suggestion. In the revision, we added a comprehensive sequence-level analysis of antisense promoters, including base composition, promoter class (TATA vs TATA-less), TATA positional distribution relative to the TSS, and de novo motif discovery with TF-motif matching.

Base composition: We computed GC% within the 500 bp regions upstream of each antisense TSS identified in our dataset (n = 1519) and, for comparison, the same window upstream of sense PCG TSSs. As our antisense TSSs were predominantly found within gene bodies, we have additionally included random 500 bp windows sampled from gene bodies as a control.

Antisense promoters had mean GC content of 33.5%, comparable to sense protein-coding promoters (32.3%) and distinct from random gene-body windows (37.8%), consistent with promoter-like composition.

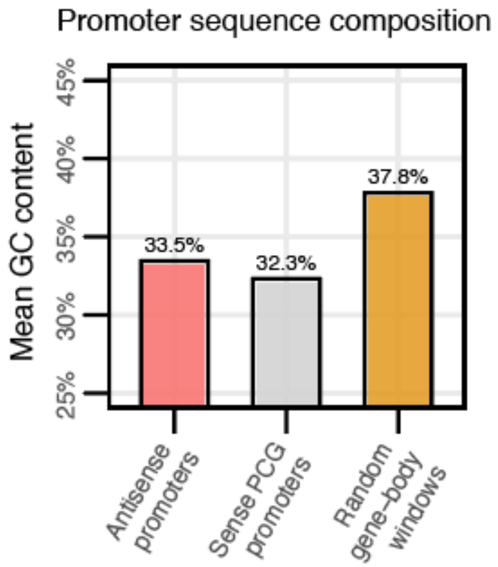

Promoter class: We scanned up to −500 bp upstream of each antisense TSS with FIMO using the TATA-box position weight matrix (PWM) obtained from the JASPAR CORE Fungi (non-redundant v2) database. Using a per-site threshold of P ≤ 4×10^-4, promoters with ≥1 hit were called as TATA-positive.

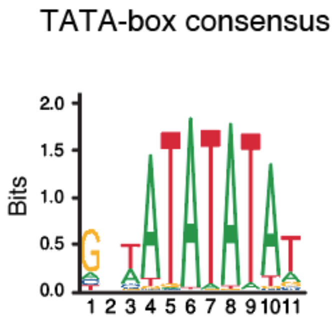

Our analysis showed that 19.2% (291/1519) of antisense promoters were TATA-positive versus 22.6% (1162/5145) of sense promoters, indicating similar class distributions.

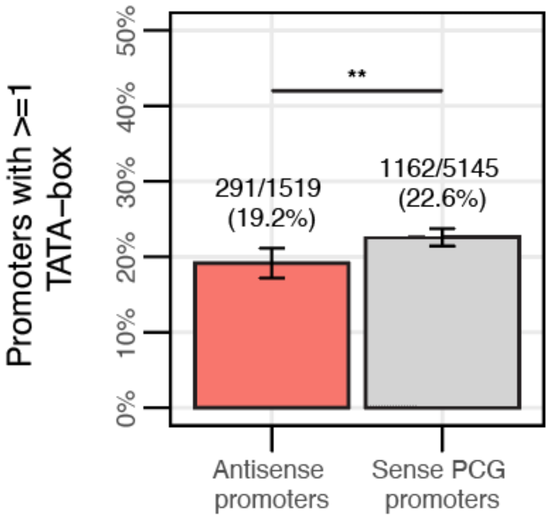

TATA positioning: For FIMO-positive sites we plotted hit-midpoint densities across −500 to 0 bp relative to the TSS (antisense vs sense).

Both showed enrichment within ~50 bp upstream of the antisense TSS, mirroring a distribution in sense promoters. Although a Kolmogorov–Smirnov test indicated a small difference (D = 0.107, P = 4.28×10^-6), we note that antisense TSSs lie within gene bodies where upstream regions are more GC-rich, reducing distal TATA frequency.

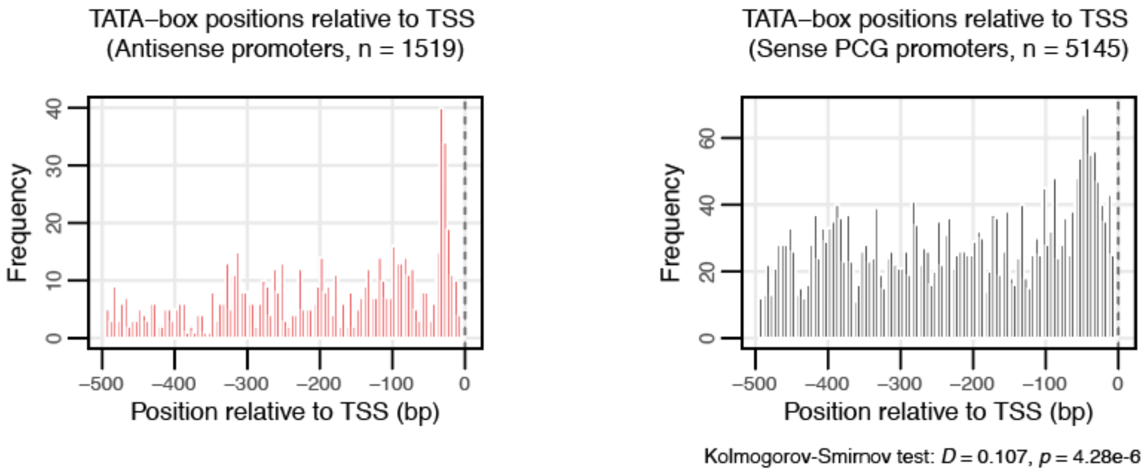

Transcription factor motifs: We performed de novo motif discovery with STREME on −500 to 0 bp sequences (width 6–15 bp; first-order Markov background from shuffled input; default E ≤ 0.05).

Seven enriched motifs were identified, each occurring between 178–758 times (Table S1).

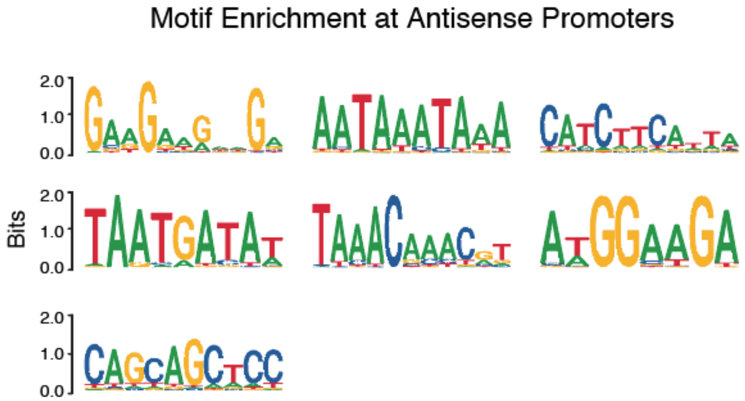

The discovered motifs were then matched to a recently published *S. pombe* TF motif atlas (Skribbe et. al., 2025) with TOMTOM (q < 0.05, minimum overlap 5 bp), reporting the top significant matches as a heatmap.

The analysis yielded significant matches for six motifs, encompassing 39% (15/38) of TFs in the study, with several motifs matching multiple TFs (e.g., Hsf1, Adn3, Sak1, Fkh2).

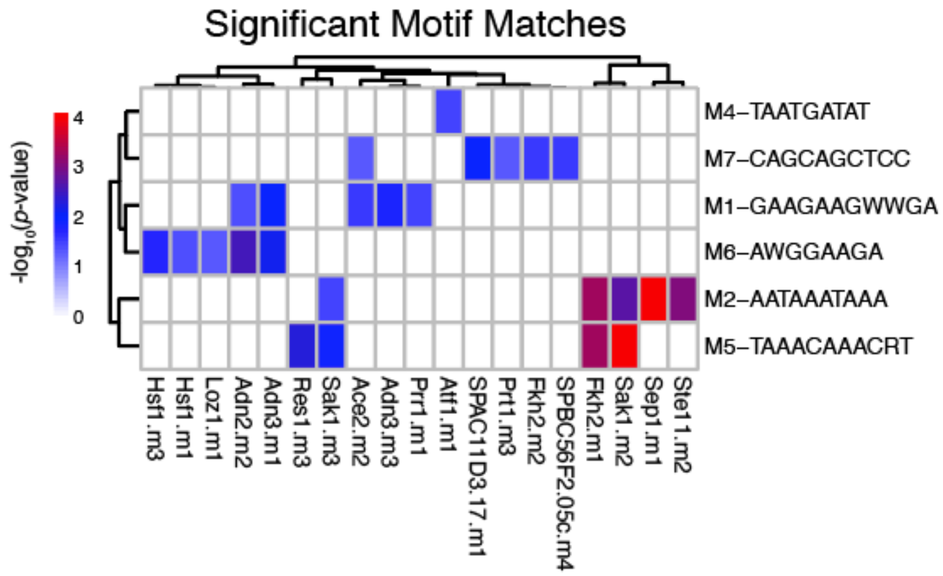

Together, these results indicate that the antisense promoters identified in this study share sequence composition, promoter class, TATA-distribution and TF-motif architecture characteristic of bona fide promoters, and they nominate specific TFs and promoter architectures that could drive antisense expression in the absence of Hrp3.

The above figures were added as new **Figure 3G**, Supplementary **Figure S5A-F** and **Table S1**. The corresponding text has been added to the results as follows (line 220–237):

‘To assess whether these regions exhibit promoter-like features, we analyzed their sequence composition and motif content. The mean GC content of antisense promoters was 33.5%, comparable to sense promoters of protein-coding genes (PCGs) in fission yeast (32.3%) and distinct from gene-body regions (37.8%; **Fig. S5A**). We next quantified the fraction of promoters containing at least one TATA-box (consensus TATAWAWR; **Fig. S5B**). 19.2% of antisense promoters contained at least one TATA-box, comparable to 22.6% among sense PCG promoters (**Fig. S5C**). The TATA-box positions were enriched within ~50 bp upstream of the As-TSS (**Fig. 3G**), mirroring the positional distribution observed for sense PCG promoters (**Fig. S5D**). *De novo* motif discovery within the 500 bp upstream of the As-TSS identified seven significantly enriched sequence motifs (**Fig. S5E**), each occurring 178–758 times in the analyzed promoter set (**Table S1**). Motif-to-TF mapping against a curated *S. pombe* TF motif atlas[1] revealed that six of the seven motifs significantly matched known TF binding specificities, collectively corresponding to 15 of the 38 TFs represented in that atlas (**Fig. S5F**). Several motifs aligned with more than one TF motif (including Hsf1, Adn3, Sak1, and Fkh2), indicating potential binding redundancy and supporting the capacity of these regions to recruit transcriptional regulators and initiate RNAPII transcription. Together, these analyses indicate that the identified antisense promoters share sequence features and core promoter architecture typical of sense promoters in fission yeast.’

Additionally, the methods have also been updated accordingly (lines 1040–1047).

Major Comment 3: The Hrp3-Prf1 interaction is demonstrated solely through recombinant overexpression and pulldown assays, which carries the risk of detecting non-physiological interactions. While the authors use mutations to verify pulldown specificity, in vivo evidence for this interaction is absent. Given that the authors cite a recent preprint demonstrating sophisticated techniques to show S. cerevisiae Chd1-Prf1 interactions, I presume standard approaches such as co-immunoprecipitation followed by mass spectrometry or Western blot were attempted. Even negative results from such experiments should be reported, as readers will likely question the physiological relevance of the interaction. Additionally, establishing the hierarchy between Hrp3, Prf1, and H2BK119Ub is crucial. While the authors show that Hrp3 ChIP-seq signal correlates with gene expression levels, the proposed Prf1-Hrp3 interaction raises questions about recruitment specificity and hierarchy. The authors mention in lines 344-345: "...the CHCT domain of Hrp3 is critical for its association with transcription elongation along the gene body..." which requires support from experimental data. Testing Hrp3 ChIP-seq in Prf1-depleted conditions would clarify how specificity is achieved and substantiate the functional importance of this interaction. As the authors have all the required strains I would estimate around 1.5-2 months for data generation and analysis.

We thank the reviewer for this thoughtful and constructive comment. We agree that *in vivo* validation and establishing pathway specificity and hierarchy are important. In the revision, we have added new experiments and analyses that directly address both points and have integrated them with our model.

These data were partially presented in the context of Reviewer 2, Comment 12 above, and are reproduced below.

Regarding the endogenous Hrp3-Prf1 interaction *in vivo* :

We have performed co-immunoprecipitation in cells expressing Prf1-FLAG and Hrp3-Myc from their endogenous loci. Whole-cell extracts were immunoprecipitated with anti-FLAG (M2) beads and probed by immunoblotting. Hrp3-Myc co-precipitated with Prf1-FLAG in two independent biological replicates (see below). The signal is modest but reproducible across replicates, consistent with a specific yet transient interaction *in vivo*.

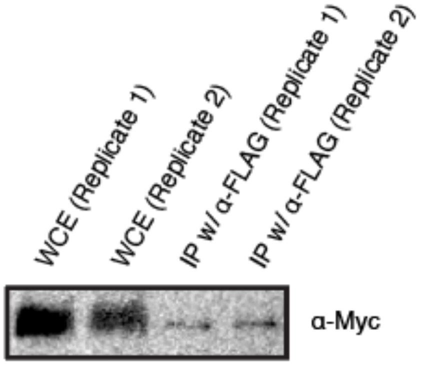

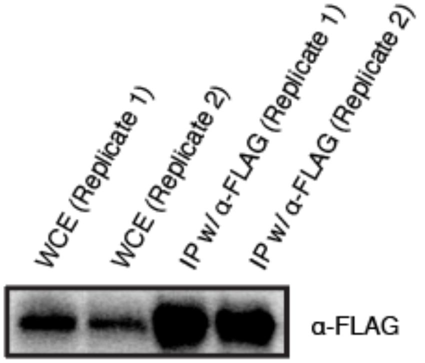

Together with our *in vitro* results, the endogenous co-IP supports a specific Hrp3–Prf1 interaction *in vivo.*

We have added our results as a new figure panel and updated the text in the results section as follows (lines 360-363):

'To validate the predicted interaction in cells, we first performed endogenous co-immunoprecipitation from strains expressing Prf1-FLAG and Hrp3-Myc. Anti-FLAG IP recovered a modest but reproducible amount of Hrp3-Myc (**Fig. S8I**), consistent with a specific but likely transient association *in vivo*.'

Regarding hierarchy and recruitment specificity of the Hrp3-Prf1 interaction:

We profiled (Myc-tagged) Hrp3 via ChIP-seq in WT and *prf1*Δ strains and analyzed metagenes, genome-wide signal distributions, and loci.

On a metagene level, Hrp3 in WT is enriched over gene bodies with a peak between the TSS and TTS. In *prf1*Δ, the profile flattens and broadens beyond the TSS and TTS, consistent with reduced association of Hrp3 with elongating gene bodies and increased association in intragenic regions.

Review COMMONS

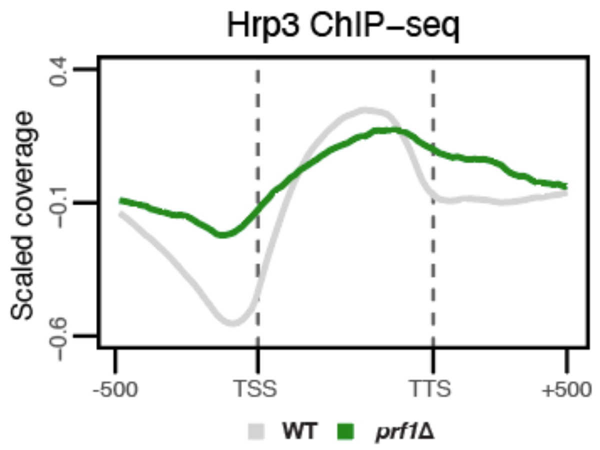

We quantified the fraction of Hrp3 signal assigned to genes versus intergenic regions and observed a shift from predominantly genic signal in WT to increased intergenic signal in *prf1*Δ (Fisher's exact test, $p < 2.2e\text{-}16$), supporting reduced gene-body targeting in the absence of Prf1.

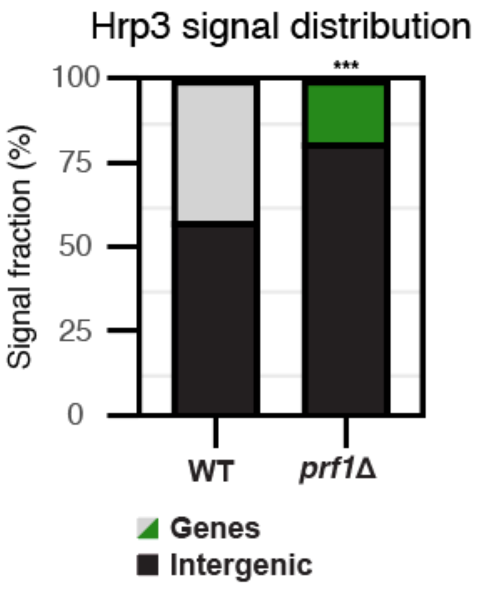

Genome browser tracks (~20 kb) of Hrp3 coverage in both strains also illustrate representative regions where specific gene-body Hrp3 signal in WT becomes more scattered and fragmented in *prf1*Δ.

Review COMMONS

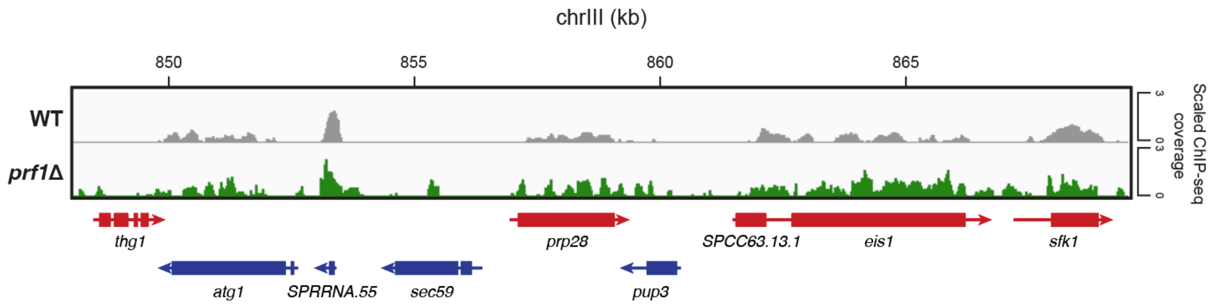

Together with the positive correlation between Hrp3 occupancy and transcription level (**Fig. 7B**), these observations support a role for Prf1 in promoting Hrp3 targeting during elongation.

To refine the pathway hierarchy, we also profiled H2BK119ub in *prf1*Δ and compared it to WT, *hrp1*Δ, *hrp3*Δ and *hrp1*Δ*hrp3*Δ. Relative to WT, *prf1*Δ exhibits a global loss of H2Bub from the TSS through the TTS that is more pronounced than in *hrp3*Δ or *hrp1*Δ*hrp3*Δ (metagene, attached).

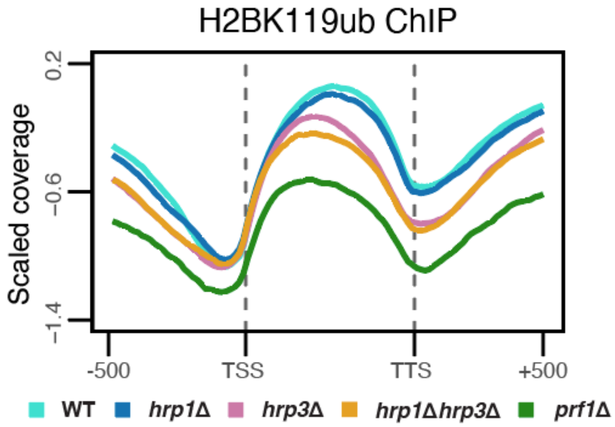

Quantification across gene-length quartiles (Q1–Q4) shows significant reductions in all bins (see response to Minor Comment 4), consistent with Prf1-dependent deposition of H2Bub on gene-body nucleosomes.

Taken together, these results support a model in which Prf1 acts upstream of Hrp3: Prf1 interacts with and promotes recruitment of Hrp3 during RNAPII elongation, facilitating efficient H2Bub deposition. H2Bub, together with readers such as FACT, cooperates with Hrp3 remodeling to establish robust gene-body nucleosome phasing that occludes cryptic promoter-like sites and represses antisense initiation. We note that Hrp3 likely retains intrinsic nucleosome-binding via its chromodomains, consistent with residual Hrp3 signal in *prf1*Δ**.**

We have added all the figures in this response as new **Figures 7C**, **7D**, **S10C** and **7E**, and revised the manuscript text to reflect this Prf1-dependent contribution to Hrp3 association with elongating gene bodies as follows (lines 391–403):

'The contrast between Hrp1 and Hrp3's genomic profiles suggested that the CHCT domain of Hrp3 may drive its co-localization to zones of active elongation. To test this, we performed Hrp3 ChIP-seq in a *prf1*Δ background, finding a marked reduction in gene body enrichment (**Fig. 7C–D; S10C**, Fisher's exact test, $p < 2.2e\text{-}16$), and establishing that Prf1 is a key determinant of Hrp3's genomic localization.
We next investigated the functional link between Hrp3, Prf1, and nucleosome positioning. We first profiled H2BK119ub in WT, *hrp1*Δ, *hrp3*Δ, *hrp1*Δ*hrp3*Δ, and *prf1*Δ (**Fig. 7E**). As expected, *prf1*Δ showed a pronounced loss of H2Bub across gene bodies. By contrast, *hrp3*Δ and *hrp1*Δ*hrp3*Δ also displayed significant, albeit less severe, reductions, whereas *hrp1*Δ alone did not (**Fig. S10D**). These results support a hierarchy in which Prf1 functions upstream of Hrp3, with the Hrp3 CHCT domain facilitating Prf1-dependent H2Bub deposition. The effect was modestly stronger at highly expressed genes (**Fig. S10E**), consistent with a tight coupling of Prf1/Hrp3 activity to transcription elongation.'

Major Comment 4: [Optional] Based on structure predictions the authors suggest that the interaction of of CHD1 and RTF1 is conserved in arabidopsis and mouse. This should be further supported by pulldown assays and also the pre-print (Reference nr. 99) should be cited as they show similar results using yeast-tow-hybrid assays.

We thank the reviewer for this suggestion. We co-expressed GST-tagged CHD1-CHCT domains from *Arabidopsis thaliana* (A.t.) and *Homo sapiens* (H.s.) with 6×His-tagged RTF1 (A.t. and H.s.) in *E. coli* and performed anti-GST pulldowns.

The Western blots show that $CHCT_{A.t.}$ robustly pulls down $RTF1_{A.t.}$, and similarly $CHCT_{H.s.}$ robustly pulls down $RTF1_{H.s.}$, but the GST-only and bead-only controls do not.

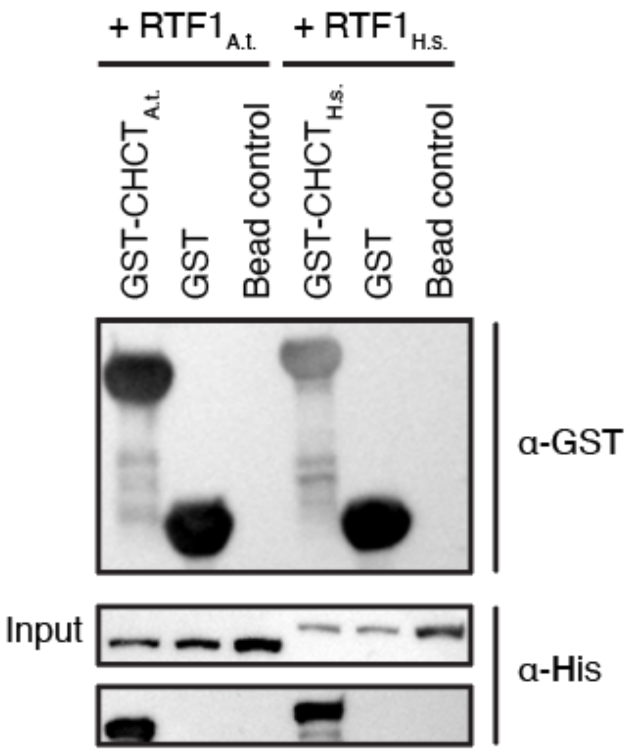

These results support our structural predictions from Alphafold that the conserved CHD1–RTF1 interaction is conserved across plants and animals in addition to fungi, strengthening the inference that the interaction is broadly conserved across eukaryotes.

We have included the results as new **Figure S8J** and added these results as follows (lines 364–366, 374-377):

‘In bacterial co-expression assays, GST-tagged Hrp3-CHCT pulled down His-tagged full-length Prf1/RTF1, and this extended to orthologs from both *Homo sapiens* and *Arabidopsis thaliana* (**Fig. S8J**), supporting a conserved interaction.’

‘Together with a recent study[94] independently reporting a similar interaction with mouse CHD1/2 and RTF1, these results demonstrate that the Hrp3/CHD1 CHCT domain mediates a conserved interaction with Prf1/RTF1 and that this interface is critical for cellular fitness under stress.’

Minor Comment 1: Figure 1B: Grouping individual panels according to different paralog groups would make the figure more accessible.

We thank the reviewer for this helpful suggestion. To enhance the accessibility and clarity of Figure 1B, we have reorganized the individual panels to group them by the different paralog groups and have clearly

labeled them accordingly. This revised layout provides a more intuitive and visually accessible representation of the data. The updated figure is included in the revised manuscript as follows:

Original:

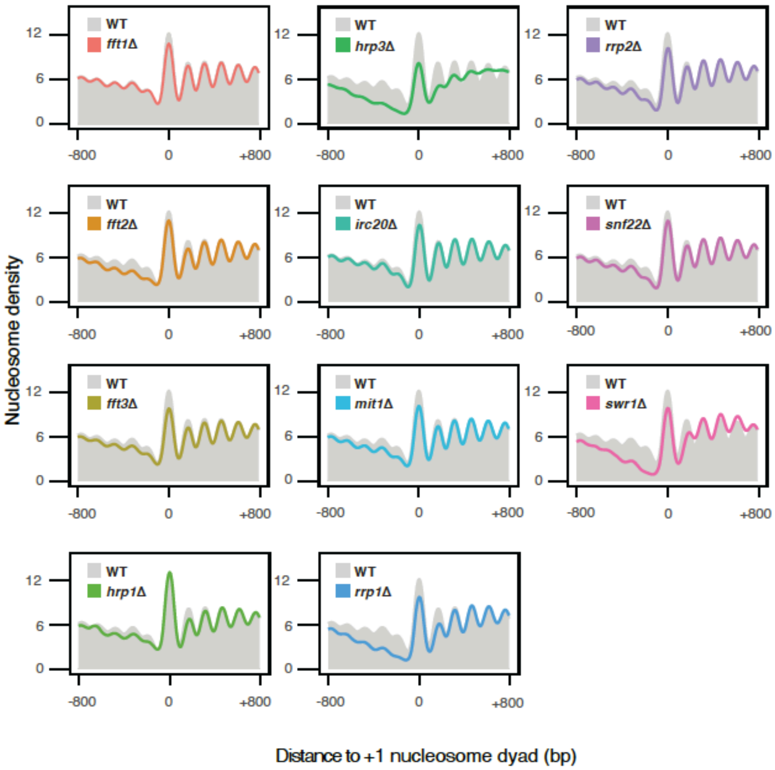

Updated:

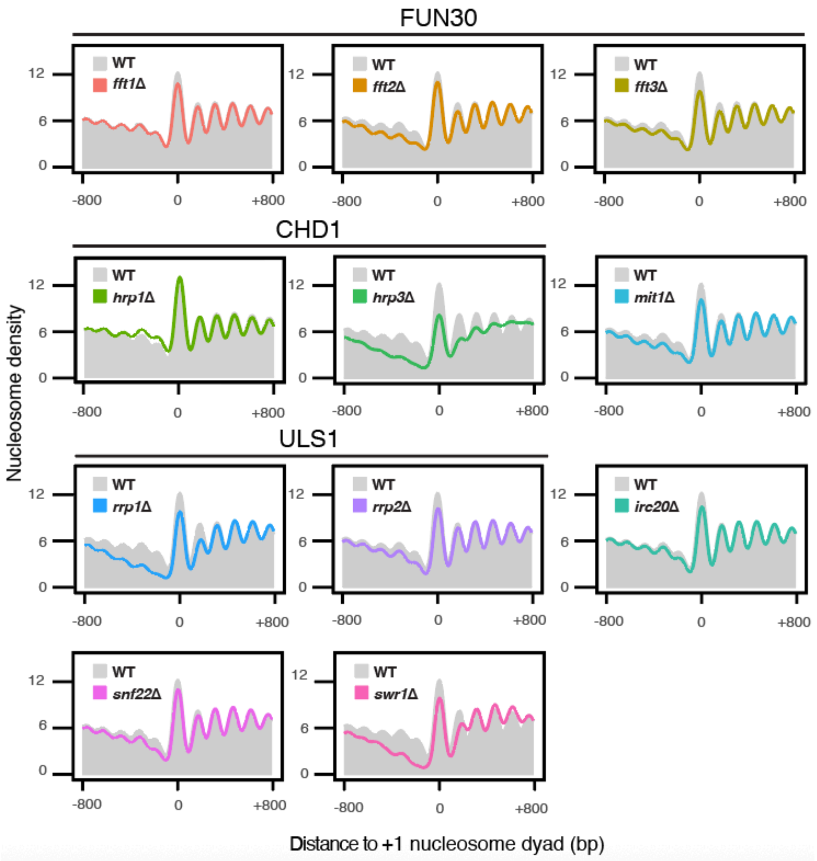

Minor Comment 2: Figure 1D: The display of antisense transcription is not accessible. Perhaps boxplots, like those in Figures 2B and 5D, would be easier to read.

We thank the reviewer for this suggestion and agree that the original display was harder to read. As the reviewer has suggested, we have revised Figure 1D to use boxplots in place of the original visualization, which improves readability and makes it clear that the *hrp3Δ* mutant shows elevated antisense transcription relative to the other remodeler mutants.

Original:

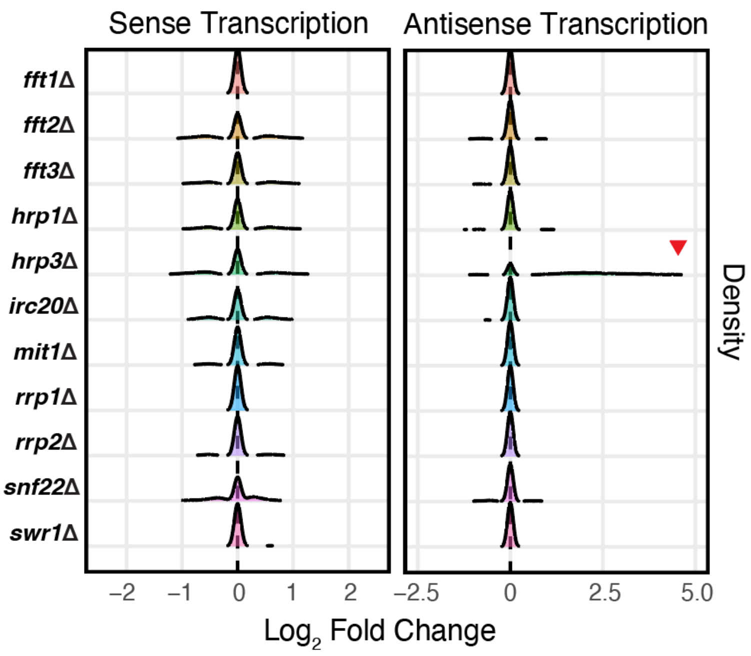
Sense Transcription
Antisense Transcription
fft1Δ
fft2Δ
fft3Δ
hrp1Δ
hrp3Δ
irc20Δ
mit1Δ
rrp1Δ
rrp2Δ
snf22Δ
swr1Δ
Density
−2
−1
0
1
2
−2.5
0
2.5
5.0
Log2 Fold Change

Updated:

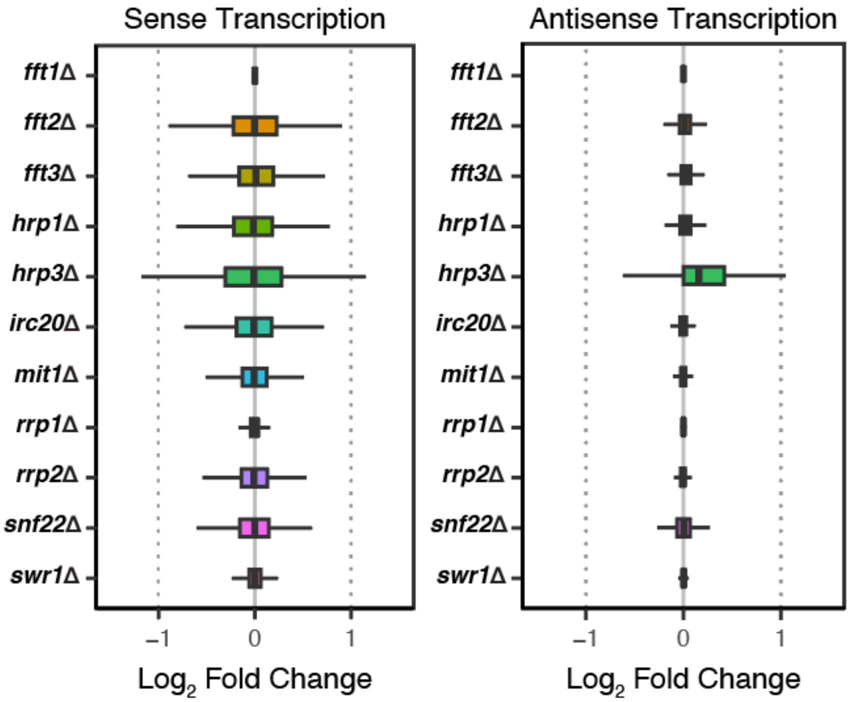
Sense Transcription
Antisense Transcription
fft1Δ
fft2Δ
fft3Δ
hrp1Δ
hrp3Δ
irc20Δ
mit1Δ
rrp1Δ
rrp2Δ
snf22Δ
swr1Δ
−1
0
1
Log2 Fold Change
Log2 Fold Change

Minor Comment 3: Line 335: The transition is abrupt and would benefit from additional explanation. Why do the authors use Rtf1 instead of Prf1 here? Consistent nomenclature would improve clarity.

We thank the reviewer for highlighting the abrupt transition and the inconsistency in nomenclature. To address this, we have added an additional section at the beginning of the paragraph to better explain our motivations for the subsequent experiments, ensuring a smoother transition from the previous section. The updated text is as follows (lines 381-386):

'We next investigated the impact of the Hrp3-Prf1 interaction on chromatin and transcriptional regulation. Prf1/RTF1 is enriched across gene bodies and is essential for recruitment of the H2B ubiquitination machinery via its histone modifying domain (HMD)[95], a crucial step in the activation of the histone chaperone FACT. We hypothesized that the Hrp3–Prf1 interaction synergizes to regulate H2Bub deposition specifically during transcription elongation, ensuring retention of nucleosomes in the wake of transcription.'

Furthermore, we have revised the nomenclature throughout the manuscript to ensure consistency and clarity. Specifically, we now use "Prf1/RTF1" when referring to the conserved protein across species and "Prf1" when specifically discussing the *S. pombe* homolog, ensuring clarity while maintaining accuracy. The changes have been implemented throughout the manuscript.

Minor Comment 4: Line 352: For the phrase "significant loss," please provide a statistical test or omit the word "significant."

We thank the reviewer for raising this point. Because the loss of H2Bub in *hrp3*Δ and *hrp1*Δ*hrp3*Δ progressively diverges from WT along the gene body, we stratified the length of gene bodies into four quartiles (Q1-Q4) and visualized per-gene signals as boxplots across the four gene-body quartile windows with statistical testing (see below). Using Welch's two-sample t-test, we observed significantly decreased signal in *hrp3*Δ and *hrp1*Δ*hrp3*Δ relative to WT beginning at Q2 and extending through Q4, consistent with the observation from the metaplot. In comparison, *prf1*Δ showed significantly decreased H2Bub across all quartiles, consistent with its function as a recruiter of the H2B ubiquitin machinery. We now present these results in a new panel (**Figure S10D**).

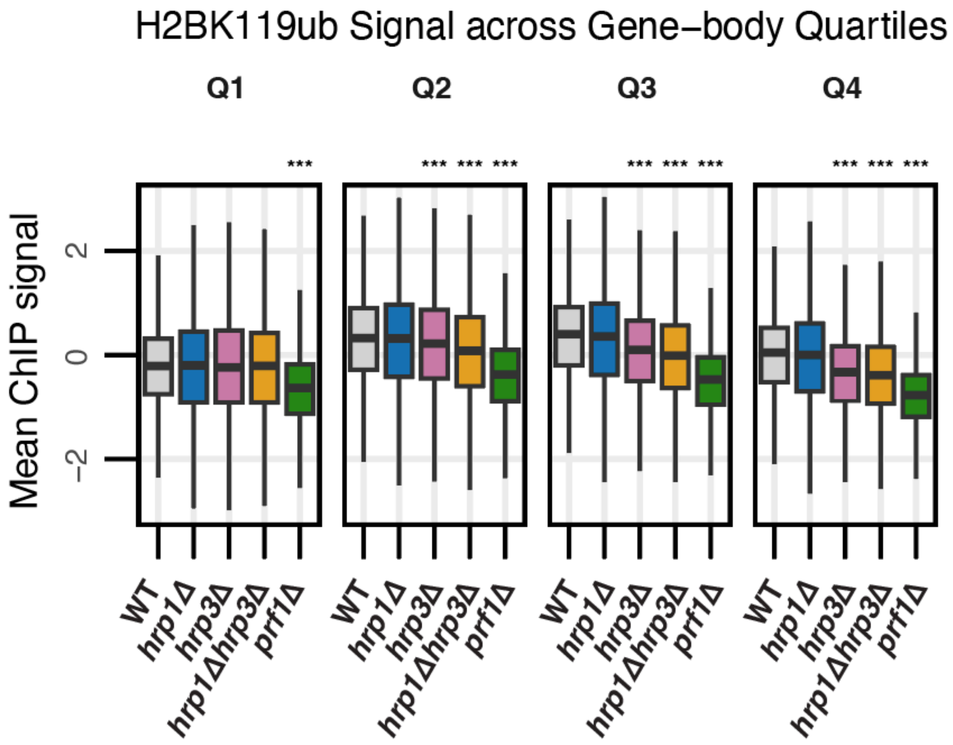

Minor Comment 5: Figure 7F: The model presented in panel F suggests that there are two parallel routes that lead to nucleosome phasing; however, the authors state in the text (lines 363-364): "further supporting the idea that Hrp3 and Prf1 act together in the same pathway to control antisense transcription." The model and the text should align better.

We thank the reviewer for pointing out this inconsistency between the model in Figure 7F and the text. To address this, we have revised the model in Figure 7F (now Figure 7H) by removing one of the parallel routes, ensuring that it now reflects the idea that Hrp3 and Prf1 act together in the same pathway to control nucleosome phasing and antisense transcription.

Original:

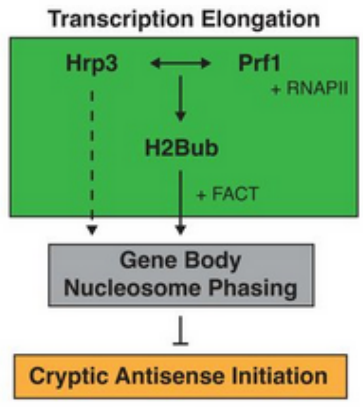

Updated:

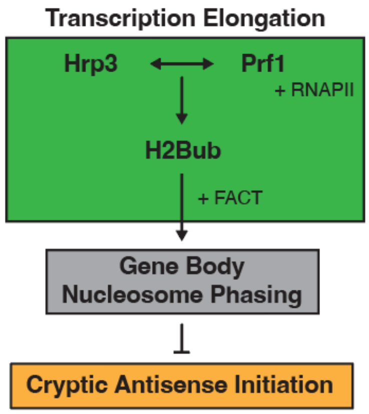

Additionally, we have expanded the Discussion to place CHD1 remodelers' nucleosome-spacing activity in broader context. These clarifications have been added to the discussion as follows (lines 478–489):

'Although our data support a model in which Prf1/RTF1 and FACT cooperate with Hrp3 to establish gene-body phasing, we do not claim that this is the only route to phasing *in vivo* or that Hrp3 lacks intrinsic spacing capacity. CHD-family remodelers can space nucleosomes *in vitro*[64,115,116], but these studies primarily report sliding-based spacing on preassembled arrays. By contrast, our *in vivo* profiles emphasize positional losses and redistributions of specific gene-body nucleosomes, which reflect not only spacing per se but also placement, stabilization, and retention on chromatin. Thus, Hrp3 may be fully capable of autonomous spacing of nucleosomes, while in cells it acts with elongation factors to position and maintain nucleosomes at critical genomic sites. This framework helps reconcile differences between *in vitro* spacing patterns[116] and *in vivo* MNase-seq profiles and underscores the need to study remodelers in their native context, where interactions with elongation factors and histone chaperones tune when and where Hrp3 operates.'
