# Supplementary material for: Nucleosome positioning shapes cryptic antisense transcription
Source: PLoS Genet. 2026 Mar 13;22(3):e1012078. doi: 10.1371/journal.pgen.1012078 (PMC13075793; doi:10.1371/journal.pgen.1012078)
Supplement: Attachment — Submitted filename: Response to Reviewers .docx [file pgen.1012078.s018.docx]

**Response to Reviewers**

Dear Editor and Reviewers,

We thank the Editor and Reviewers for their careful evaluation of our manuscript, *“*Nucleosome Positioning Shapes Cryptic Antisense Transcription.*”* We appreciate the additional comments and suggestions provided during the second round of review, which have helped further clarify and strengthen the manuscript. Below, we respond to each point in turn. Reviewer comments are shown in green, followed by our responses in plain text.

**Reviewer #1**

The only experiment requiring further clarification is the co-immunoprecipitation followed by western blotting to demonstrate the Hrp3–Prf1 interaction in vivo (Reviewer 3, Major Comment 3; Reviewer 2, Comment 12). To interpret this experiment, it is necessary to include a FLAG pulldown followed by anti-Myc western blot in cells where Prf1 is not FLAG-tagged. Given the weak interaction observed, background/non-specific signal from Hrp3 is a technical concern. Depending on the results, the authors should either include this control or adjust their claims regarding an in vivo interaction accordingly.

We thank the reviewers for their constructive comments regarding the co-immunoprecipitation experiment and the need for appropriate negative controls. To address these concerns, we repeated the *in vivo* anti-FLAG co-immunoprecipitation using a Prf1-FLAG strain alongside an otherwise isogenic no-FLAG control. Hrp3-Myc was reproducibly detected in FLAG immunoprecipitates but was not enriched in the untagged control or the Protein A-agarose bead control. Anti-FLAG immunoblotting confirmed efficient and specific recovery of Prf1-FLAG. These data support a specific in vivo association between Prf1 and Hrp3.

We have updated the figure (**Figure S9I**) and legend in the supporting information accordingly to reflect the inclusion of these controls.

The updated text is as follows:

‘(I) Endogenous co‑immunoprecipitation of Hrp3 with Prf1 from *S. pombe*. Whole‑cell extracts (Input) were prepared from strains expressing Prf1‑FLAG and Hrp3‑Myc from their native loci, alongside an otherwise isogenic no-FLAG control strain. Anti-FLAG immunoprecipitations (IP) were analyzed by immunoblotting with anti-Myc to detect co-precipitating Hrp3-Myc (top) and anti-FLAG to confirm specific recovery of Prf1-FLAG (bottom). Protein A–agarose beads were included as a matrix control.’

**Reviewer #3**

Point 5.

The explanations of Figures 1D, 2B and 3A and 3B in the two paragraphs are clear and well described. I think these explanations, or a summary of them, should be included in the text to prevent readers from having similar doubts to those I had when reading the manuscript.

We thank the reviewer for this suggestion and agree that these clarifications should be included in the manuscript. We have now added explicit explanations in the Results section to guide the reader in interpreting these datasets. Specifically, we clarify that gene-level mRNA-seq summaries in Fig. 1D primarily reflect effect size rather than statistical significance, explain why *hrp1*Δ shows a clearer antisense signal in PRO-seq than in mRNA-seq due to assay sensitivity to initiation-proximal events, and note that sense transcription profiles remain broadly comparable across strains despite minor visual differences between datasets. These additions are intended to prevent similar reader confusion.

Updated text:

(lines 157-160)

‘At the transcriptome-wide level, this modest effect is reflected by a small median shift with a narrow interquartile range (**Fig. 1D**), indicating a limited effect size despite statistical significance in pairwise comparisons (**Fig. 2B**).’

(lines 194-196)

‘Despite minor visual differences between datasets, mean sense transcription profiles are broadly comparable across strains, consistent with the minimal changes observed by mRNA-seq (**Fig. 2A**).’

(lines 196-199)

‘Because PRO-seq measures nascent transcription initiation rather than steady-state RNA abundance, it is particularly sensitive to initiation-proximal antisense events.’

Point 10.

The negative control is still absent. The new qPCR assay histogram should show eight columns: the mRNA level of the wild-type ura4 gene (positive control), the level in the strain where its promoter has been replaced by that of adh1, the level in the strain where the ura4 promoter has been deleted (negative control), and the level of the five putative promoters to be tested. The results should be presented by normalization of the ura4 level in each strain relative to act1 without the need to refer them to the strain where the ura4 promoter has been deleted. This would enable the promoter efficiency in the different strains to be compared directly.

We thank the reviewer for this helpful suggestion regarding normalization and data presentation. We have revised the figure to display *ura4* expression normalized directly to *act1* (2^ΔCq^) for all constructs in a single plot.

Because the wild-type S. pombe 972h^-^ background strain in the lab used for this reporter assay carries a *ura4-D18* allele, a wild-type *ura4* promoter driving the endogenous *ura4* locus is not available in this experimental context. Instead, the S. pombe *adh1* promoter is used as the positive control, and a promoter-deletion strain as a negative control, alongside the five candidate promoter sequences.

We believe this revised visualization improves clarity and enables direct comparison of promoter strengths across constructs.

The updated **Figure 3I** is as follows:

The corresponding legend have been updated accordingly (lines 652-659):

‘Bar plot showing expression of the *ura4* gene relative to the *act1* gene by RT-qPCR (2⁻^ΔCq^) under five different antisense promoters (from H). The *adh1* promoter serves as a positive control, while a promoter-deletion strain (null) serves as a negative control. Bars represent mean values from three biological replicates; points indicate individual replicates. The dotted line denotes the mean expression level of the promoter-deletion negative control. Statistical significance was determined using Welch’s t-tests comparing each promoter construct to the promoter-deletion negative control, with Holm correction for multiple testing. Asterisks indicate statistical significance: p < 0.05 (*), p < 0.01 (**), p < 0.001 (***), n.s. (not significant).’

Point 11.

I agree that a detailed analysis of the role of hrp1 and hrp3 in meiosis is beyond the scope of this work. However, without further analysis, it is impossible to determine whether defects in meiosis are caused by antisense-mediated deregulation of meiosis-specific genes, given that these genes only account for a fraction of all genes that are deregulated in mutants. Furthermore, this possibility is inconsistent with the observation that the hrp1Δ mutant exhibits more severe alterations to meiosis despite displaying significantly lower levels of antisense expression than the hrp3Δ mutant (Fig 1D and 2A).

We agree with the reviewer that our data do not support attributing meiotic defects specifically to antisense-mediated deregulation of meiosis-specific genes. We have therefore revised the Results section to clarify that Hrp3-dependent antisense repression contributes to the regulation of convergently nested meiotic genes during vegetative growth, but represents one component of a broader regulatory landscape in CHD remodeler mutants.

With respect to the apparent discrepancy between antisense levels and meiotic phenotypes, we note that our data indicate substantial functional divergence between Hrp1 and Hrp3 across multiple assays, including antisense transcription profiles and nucleosome organization (**Fig. 1B-D**), chromatin localization and H2Bub distribution (**Fig. 7A, 7E**), stress sensitivity (**Fig. 5B**), and expression timing during meiosis (**Fig. 4F**). These observations suggest that the two paralogs are not simply distinguished by the magnitude of antisense deregulation alone. Accordingly, the finding that *hrp1*Δ exhibits pronounced meiotic defects despite comparatively lower antisense levels does not necessarily constitute a contradiction, but rather indicates that the quantitative efficiency of meiotic progression likely integrates multiple molecular pathways.

To reflect this more nuanced interpretation, we have updated the Results as follows (lines 309-317):

‘Notably, *hrp1*Δ exhibited pronounced meiotic defects despite comparatively lower levels of antisense transcription, suggesting that meiotic progression does not follow a simple quantitative relationship with global antisense deregulation. These observations indicate that while Hrp3-dependent antisense repression contributes to the regulation of convergently nested meiotic genes during vegetative growth, meiotic progression likely reflects additional molecular pathways or paralog-specific properties of CHD remodelers. Thus, CHD remodeler activity represents a context-dependent regulatory layer within a broader chromatin and transcriptional framework coordinating nested and meiotic gene programs in fission yeast.’

We have also updated the Results subheading (lines 282-283) to better reflect an associative rather than strictly causal interpretation:

Original:

‘Hrp3-Mediated Antisense Transcription Regulates Meiotic and Nested Gene Expression in *S. pombe*’

Updated:

‘Hrp3-Mediated Antisense Repression Links Nested Gene Architecture to Meiotic Gene Expression in *S. pombe*’

Point 12.

In vivo co-immunoprecipitation experiments show that Hrp3-Myc is faintly detected after Prf1-FLAG immunoprecipitation. This experiment, however, lacks a negative control using either agarose beads or agarose beads coupled to an unrelated antibody, which would show that the a-Myc signal is specific and not due to background. To provide further confirmation, the authors could carry out reverse co-immunoprecipitation, first with Hrp3-Myc and then with Prf1-FLAG.

We thank the reviewers for their constructive comments regarding the co-immunoprecipitation experiment and the need for appropriate negative controls. This point was also raised by Reviewer 1 (see above, reproduced below).

To address these concerns, we repeated the in vivo anti-FLAG co-immunoprecipitation using a Prf1-FLAG strain alongside an otherwise isogenic no-FLAG control. Hrp3-Myc was reproducibly detected in FLAG immunoprecipitates but was not enriched in the untagged control or the Protein A-agarose bead control. Anti-FLAG immunoblotting confirmed efficient and specific recovery of Prf1-FLAG. These data support a specific in vivo association between Prf1 and Hrp3.

We have updated the figure (**Figure S9I**) and legend in the supporting information accordingly to reflect the inclusion of these controls.

The updated text is as follows:

‘(I) Endogenous co‑immunoprecipitation of Hrp3 with Prf1 from *S. pombe*. Whole‑cell extracts (Input) were prepared from strains expressing Prf1‑FLAG and Hrp3‑Myc from their native loci, alongside an otherwise isogenic no-FLAG control strain. Anti-FLAG immunoprecipitations (IP) were analyzed by immunoblotting with anti-Myc to detect co-precipitating Hrp3-Myc (top) and anti-FLAG to confirm specific recovery of Prf1-FLAG (bottom). Protein A–agarose beads were included as a matrix control.’

Point 13.

The new experiment in liquid medium shows that after 15 hours of growth, hrp1∆ hrp3∆ prf1∆ cells reach a lower cell density than single prf1∆ or double hrp1∆ hrp3∆ mutants. The authors should measure and represent the optical density (OD) at regular intervals over a 15-hour period, starting from an identical initial OD. This straightforward experiment would yield more detailed information about the growth rates of the four strains.

We thank the reviewer for this suggestion. We agree that time-resolved growth curves can provide additional information on growth kinetics. However, our intention with this experiment was not to compare growth rates, but to assess the relative severity of growth impairment across genotypes under a single, controlled condition.

We have revised the Results text to clarify that our conclusions are based on endpoint cell density rather than growth kinetics. Given that Prf1 is known to have essential roles beyond its cooperation with Hrp3, and that growth phenotypes are not a central focus of this study, we believe that the current quantitative data sufficiently support our conclusions without requiring additional time-course measurements.

The updated text is as follows (line 441-443):

Original:

Notably, the *prf1*Δ and *hrp1*Δ*hrp3*Δ*prf1*Δ mutants displayed stronger growth defects than the *hrp1*Δ*hrp3*Δ mutant, indicating that Prf1 has additional essential roles beyond its cooperation with Hrp3 in cells [99] (**Fig. S10H**).

Updated:

Notably, the *prf1*Δ and *hrp1*Δ*hrp3*Δ*prf1*Δ mutants reached a lower cell density at endpoint compared to the *hrp1*Δ*hrp3*Δ mutant, indicating that Prf1 has additional essential roles beyond its cooperation with Hrp3 in cells [99] (**Fig. S10H**).
